# Neuron-derived neurotrophic factor-positive interneurons: a cellular target for anti-seizure therapies

**DOI:** 10.1016/j.ebiom.2026.106362

**Published:** 2026-07-21

**Authors:** Amy Richardson, Marion S. Mercier, Yoshiteru Shimoda, Robert T. Graham, Marco Leite, Alejandro Garcia, Maud Muller, Suraya A. Bond, Maria Grozdanova, Qimin Wu, Andreas Lieb, Dimitri M. Kullmann, Vincent Magloire

**Affiliations:** a UCL Queen Square Institute of Neurology, University College London, London, WC1N 3BG, United Kingdom

**Keywords:** NDNF+ interneurons, Epilepsy, Inhibition, Anti-seizure therapies

## Abstract

**Background:**

Failure of epilepsy pharmacotherapy is common, justifying efforts to identify optimal cell targets for advanced therapies. Genetic manipulations of the inhibitory system in principle offer a finely tuned intervention that cannot be achieved with a broad reduction of pyramidal neuron excitability. In this context, neuron-derived neurotrophic factor-expressing (NDNF+) interneurons evoke long-lasting GABA_A_ and GABA_B_ receptor-mediated inhibition. However, their contribution to restraining hyperexcitability during epileptiform activity remains unexplored.

**Methods:**

To address this, we employed calcium imaging, optogenetics, and chemogenetics in an NDNF-Cre mouse line to investigate how NDNF+ interneurons influence cortical and hippocampal hyperactivity and spontaneous seizures.

**Findings:**

We show that NDNF+ interneurons are actively recruited during interictal spikes and focal seizures, although more slowly than parvalbumin-positive interneurons (mean difference: 2.8 s, p = 0.023). Optogenetic hyperpolarisation of NDNF+ interneurons exacerbates epileptic discharges (normalised seizure-duration mean difference: 0.23, p = 0.0474), whereas their depolarisation suppresses focal seizures, even when the optogenetic stimulus is delayed by several seconds from seizure onset (normalized seizure-duration mean difference: −0.27, p = 0.03). This effect is largely mediated by GABA_B_ receptors. Additionally, chemogenetic depolarisation of NDNF+ interneurons has an important anti-seizure effect in the hippocampus both *ex vivo* (normalised seizure-duration mean difference: −0.92, p < 0.001) and *in vivo*.

**Interpretation:**

Collectively, these findings establish NDNF+ neurons as key regulators of both cortical and hippocampal excitability even during epileptic activity, and identify them as promising cellular targets for the development of anti-seizure therapies.

**Funding:**

Epilepsy Research UK, Wellcome, the Medical Research Council, the Gatsby Charitable Foundation, and The Rosetrees Trust.

**Research in context**

**Evidence before this study**

Seizures arise from a failure of the inhibitory system to restrain a surge of neuronal hyperactivity that invades cortical structures. Pharmacotherapy frequently fails, and refractory focal epilepsy is a major unmet need. Hence, new genetic therapies are being developed, which involve either a reduction of pyramidal neuron excitability or an increase in interneuron-mediated inhibition. Whilst interfering with excitatory neurons has had some success, it has the potential to disturb normal cortical processing. Manipulating the inhibitory system represents an attractive alternative for a more fine-tuned intervention. In this context, we previously showed that the anti-seizure effects of the main classes of GABAergic interneurons fade rapidly, limiting their value as cellular targets for advanced therapies.

**Added value of this study**

We now show that neuron-derived neurotrophic factor-positive (NDNF+) interneurons support sustained inhibition during seizures, consistent with GABA_B_ receptor activation and an unusual form of slow GABA_A_ signalling. These findings provide important insights into an unexplored aspect of inhibitory restraint and highlight NDNF+ cells and GABA_B_ signalling as complementary and promising targets for anti-seizure therapies.

**Implications of all the available evidence**

Epilepsy affects approximately 1% of the population, and in ∼30% of cases, seizures are refractory to drug treatment. The only option for people with refractory focal epilepsy to achieve seizure freedom is destructive and irreversible surgery with the potential for life-long sequelae. There is therefore a very real need to identify alternative treatments. Here, we present evidence for NDNF+ interneurons acting through GABA_B_ receptors as a candidate therapeutic target to prevent seizures, although further work is needed to assess its translational potential.

## Introduction

Epilepsy affects ∼1% of the population, and in ∼30% of cases, seizures are refractory to drug treatment. Pharmacoresistant epilepsy is associated with high rates of comorbidities and increased mortality, and represents an enormous burden to society, with a cost estimated at ∼14 Billion Euros annually in Europe.^1^ Importantly, rates of pharmacoresistance have not changed in 30 years despite the introduction of over 15 new anti-seizure drugs,^2^ emphasising the need to find new approaches for understanding and treating this devastating disease. New genetic approaches are thus being developed to prevent focal seizures,^3^^,^^4^ which involve either a reduction of pyramidal neuron excitability or an increase in interneuron-mediated inhibition. Whilst the first strategy has had some success, interfering with excitatory neurons is a delicate operation with the potential to greatly disturb normal cortical processing. Manipulating the inhibitory system, on the other hand, though complex due to the diversity of interneurons, represents an attractive alternative for a subtler and more fine-tuned intervention.

Cortical and hippocampal inhibition plays a pivotal role in regulating microcircuit excitability under physiological conditions through the coordinated action of four main interneuron classes: parvalbumin-positive (PV+), somatostatin-positive (SOM+), vaso-active intestinal polypeptide-positive (VIP+) interneurons, and a fourth class, mainly comprising neurogliaform (NGF) neurons, distinguished by the expression of neuron-derived neurotrophic factor (NDNF+).^5^, ^6^, ^7^, ^8^, ^9^, ^10^ However, the contribution of each population to restraining excitation during excessive or paroxysmal activity, such as epileptiform discharges, is less clear. Whilst it is established that a failure of inhibition underlies seizure generation and propagation into new territories,^11^, ^12^, ^13^, ^14^, ^15^, ^16^ which interneuron subtypes prevent (or promote) hyperexcitability, and by which mechanisms, is still incompletely understood. The identification of distinct molecular markers, combined with advances in opto- and chemogenetic tools as well as cellular imaging, has revealed that the inhibitory control exerted by different interneuron subtypes varies significantly during epileptic activity.

For instance, PV+ cells, whilst being recruited very early during epileptic discharges,^12^^,^^13^^,^^17^, ^18^, ^19^, ^20^ can either suppress or promote cortical and hippocampal activity depending on when and where they are activated relative to the hyperexcited pyramidal neuron network.^19^^,^^21^, ^22^, ^23^, ^24^, ^25^, ^26^, ^27^, ^28^, ^29^, ^30^, ^31^, ^32^ In contrast, SOM+ interneurons are activated later during seizure activity^13^^,^^18^, ^19^, ^20^ and generally decrease network excitability when optogenetically stimulated, although this effect can wane.^19^^,^^21^^,^^22^^,^^29^^,^^31^, ^32^, ^33^ Few studies have examined the role of VIP+ interneurons in restraining excitation during epileptic activity, but *in vivo* optogenetic inhibition of VIP+ cells has been shown to increase seizure threshold^19^ and silencing their synaptic activity has anti-seizure effects,^34^ suggesting an overall reduction in network excitability. Conversely, chemogenetic activation of VIP+ interneurons in an *ex vivo* hippocampal model of epileptiform discharges produced no detectable effect.^33^ The role of NGF (NDNF+) neurons in this context remains largely unexplored. These neurons provide long-range feedforward inhibition and exhibit unusual synaptic properties, including a high density of GABA release sites along their profuse axons and a relatively wide synaptic cleft.^35^ These features enable them to signal at least in part via “volume transmission”.^35^ Characterised by the release of large GABA clouds into the extracellular space, this mechanism allows a single NGF cell action potential to induce a long-lasting inhibitory response in pyramidal neurons via both long-lasting GABA_A_ (GABA_A, slow_) and GABA_B_ receptor-mediated signalling.^7^^,^^35^^,^^36^ NGF cells thus powerfully regulate dendritic excitability both in the neocortex and hippocampus.^6^^,^^10^^,^^35^^,^^37^, ^38^, ^39^, ^40^, ^41^, ^42^ Beyond these signalling properties, NGF neurons exhibit a high connection probability across cell types and layers^36^^,^^43^^,^^44^ and form a syncytium through gap-junction coupling, not only among themselves but also with other interneurons.^9^^,^^45^ This enables them to modulate the excitability of extensive cortical territories. In addition, they express neuropeptide Y (NPY), which can exert anti-seizure effects,^46^^,^^47^ and exhibit atypical persistent barrage firing in the absence of synaptic inputs, a mechanism thought to counteract over-excitation.^44^^,^^48^^,^^49^ The lack of identified molecular markers specific to NGF interneurons has, however, until recently^6^, ^7^, ^8^ hindered investigation of their roles in states of cortical and hippocampal circuit hyperactivity such as epilepsy.

Here, we leverage a recently generated NDNF-Cre mouse line, combined with calcium imaging, optogenetics and chemogenetics to investigate the role of NDNF+ NGF interneurons in controlling hyperexcitability and spontaneous seizures in multiple *ex vivo* and *in vivo* cortical and hippocampal epilepsy models.

## Methods

### Animals

Mice were housed under a 12 h:12 h light–dark cycle and had access to food and water *ad libitum*, and were randomly allocated to treatment groups for all experiments.

Heterozygous and homozygous NDNF-Cre mice (B6.Cg-Ndnf^tm1.1(folA/cre)Hze^/J; RRID:IMSR_JAX_028536, total number: 121 mice) were locally injected with adeno-associated viral vectors carrying floxed genes of interest (PV::Cre (B6; 129P2-Pvalb^(tm1(cre)Arbr)^/J mice; RRID:IMSR_JAX_008069, total number: 29 mice) and SOM::Cre (B6N.Cg-Sst^tm2.1(cre)Zjh/J^; RRID:IMSR_JAX_018973, total number: 13 mice) mice were either crossed with Ai32 mice (B6; 129 S-Gt(ROSA)26Sor^tm32(CAG-COP4^∗^H134R/EYFP)Hze/J^; RRID:IMSR_JAX_012569) for conditional expression of EYFP-tagged ChR2 in PV+ and SOM+ target interneurons, or locally injected with adeno-associated viral vectors.^21^

### Ethics

All experimental procedures were carried out in accordance with the Animals (Scientific procedures) Act 1986 under project licences PAF2788F5 and PP3944290, were approved by the institutional Animal Welfare and Ethical Review Body, and complied with the ARRIVE guidelines.

### Plasmid construction

#### Adeno-associated virus (AAV)-mDlx-FLEX-hM3D(Gq)-mCherry

The pAAV-mDlx-FLEX-hM3Dq-mCherry transfer plasmid was created by replacing the hsyn promoter in the original plasmid pAAV-hsyn-DIO-hM3D(Gq)-mCherry (RRID: Addgene_44361) with an mDlx promoter from the original plasmid pAAV-mDlx-GCaMP6F-Fishell-2 (RRID: Addgene_83899). This was achieved by inserting flanking *MluI* and *SalI* restriction sites on either side of the mDlx promoter using the primers: Fwd—GATACGCGTctatacactcacagtggtttgg (*MluI*) and Rev–GATGTCGACctgtggagagaaaggc (*SalI*). Following PCR amplification of the mDlx promoter fragment containing the restriction sites, both the fragment and the pAAV-hsyn-DIO-hM3D(Gq)-mCherry plasmid were digested with MluI (NEB; R3198S) and SalI (NEB; R3138S). The DNA was then purified (PureLink PCR Micro kit; K310050) and the fragment ligated into the hM3D(Gq) backbone using a QuickLigation kit (NEB; M2200). Following transformation, DNA was purified (PureLink HiPure Plasmid Filter Kit; K210014) and sequenced to confirm correct insertion.

#### pAAV-mDlx-FLEX-mCherry

The pAAV-mDlx-FLEX-mCherry control plasmid was created by removing hM3Dq transgene from the pAAV-mDlx-DIO-hM3Dq-mCherry plasmid using Q5 site-directed mutagenesis (NEB E0554S) with the primers Fwd—GGTGGCGCTAGCATAACTT and Rev—GCCACCATGGTGAGCAAG. Following PCR amplification and transformation the DNA was purified (Macherey–Nagel; ThermoFisher; 11932492) and sequenced to confirm the correct deletion.

#### pAAV-mDlx-FLEX-Chronos-GFP and pAAV-mDlx-FLEX-ArchT-GFP

mDlx promoters replaced EF1α and CAG in the original plasmids pAAV-EF1α-FLEX-Chronos-GFP (RRID: Addgene_62725) and pAAV-CAG-FLEX-ArchT-GFP (RRID: Addgene_28307), respectively. The mDlx promoter placed into the Chronos-GFP backbone was taken from the original plasmid (RRID: Addgene_83899) using the following promoters to insert *PacI* and *EcoRI* restriction sites; Fwd- GATTTAATTAActatacactcacagtggtttgg and Rev—GATCGAATTCcctgtggagagaaaggc. Both the mDlx promoter fragment and the pAAV-EF1α-FLEX-Chronos-GFP were digested using PacI (NEB; R0547S) and EcoRI (NEB; R0101S). The DNA was then purified (PureLink PCR Micro kit; K310050) and the fragment ligated into the backbone using a QuickLigation kit (NEB; M2200). Following transformation, DNA was purified (PureLink HiPure Plasmid Filter Kit; K210014) and sequenced to confirm the correct insertion. The mDlx promoter was amplified from the pAAV-mDlx-FLEX-mCherry plasmid and inserted into the pAAV-FLEX-ArchT-GFP backbone using a NEB HiFi DNA assembly protocol. The primers used to amplify the mDlx promoter sequence were Fwd-atgttcccatagtaacgccaGTCTATACACTCACAGTGG; Rev-atggggatccaattctttgccTGTGGAGAGAAAGGCAAAG. Those used to amplify the backbone sequence were Fwd- GCAAAGAATTGGATCCCC; Rev—TGGCGTTACTATGGGAAC. Following amplification and NEB HiFi DNA assembly, DNA was transformed and midiprepped (Macherey–Nagel; ThermoFisher; 11932492), before sequencing to confirm correct insertion of the mDlx promoter. All plasmids listed above were fully sequenced before AAV production by VectorBuilder.

### Viral vectors

The complete list of AAVs as well as their specific use for the present study are given in Table 1.

**Table 1:** Viral constructs, kainate and injection sites (V1: primary visual cortex, S1BF: primary somatosensory cortex, barrel field).

Virus/substance	Coordinates	Volume per site	Dilution	Source
AAV9-hSyn-FLEX-GCaMP6f	**V1** AP −1.8 & −3.8 mm ML 2 mm DV −0.4 & −0.2 mm	150 nL	1:2	RRID: Addgene 100833 (Stock titre: 2.2 × 1013 GC/mL)
	**S1BF** AP 0 & −2 mm ML 3 mm DV −0.3 & −0.2 mm	150 nL	1:2	
AAV9-mDlx-FLEX-Chronos-GFP	**V1** AP −1.8 & −3.8 mm ML 2 mm DV −0.4 & −0.2 mm	150 nL	1:2	In house and VectorBuilder (Stock titre: 1 × 10^13^ GC/mL)
AAV9-mDlx-FLEX-ArchT-GFP	**V1** AP −1.8 & −3.8 mm ML 2 mm DV −0.4 & −0.2 mm	150 nL	1:4	In house and VectorBuilder (Stock titre: 3.15 × 10^13^ GC/mL)
AAV9-mDlx-FLEX-mCherry	**V1** AP −1.8 & −3.8 mm ML 2 mm DV −0.4 & −0.2 mm	150 nL	1:2	In house and VectorBuilder (Stock titre: 2.7 × 10^13^ GC/mL)
	**S1BF –** AP 0 & 2 mm ML 3 mm DV −0.3 & −0.2 mm	150 nL	1:2	
	**Hippocampus** AP −3.1 mm ML 3.12 mm DV −2.5 mm	150 nL	1:4	
AAV9-mDlx-FLEX-hM3Dq-mCherry	**V1** AP −1.8 & −3.8 mm ML 2 mm DV −0.4 & −0.2 mm	150 nL	1:2	In house and VectorBuilder (Stock titre: 1 × 10^13^ GC/mL)
	**S1BF** AP 0 & 2 mm ML 3 mm DV −0.3 & −0.2 mm	150 nL	1:2	
	**Hippocampus** AP −3.1 mm ML 3.12 mm DV −2.5 mm	150 nL	1:4	
Kainic acid	**Dorsal hippocampus** AP −2.8 mm ML 3 mm DV −2 mm	Male: 140 nL Female: 80 nL	1:3	PubChem CID 10255, Tocris #7065 (Stock concentration 20 mM)

### Surgical procedures

#### Viral vector injections

Mice of both sexes (2–6 months old) were anaesthetised with 5% isoflurane, and transferred to a stereotaxic frame (David Kopf Instruments Ltd, USA) after shaving the fur on the head. Animals were injected with buprenorphine (0.1 mg/kg, s.c. PubChem CID 644073, Vetergesic) and Meloxicam (2 mg/kg, s.c. PubChem CID 54677470, Metacam) for pain relief. Core temperature was maintained using a thermoblanket, and anaesthesia was maintained with 1.5–2.5% isoflurane. After confirming loss of the pedal reflex, an incision was made to the scalp and burr holes were drilled at different locations depending on the type of experiment (Table 1). For cortical injections, the tip of a 34-gauge bevelled needle (12°, 25 mm length; Esslab Ltd., UK) was inserted at an angle of 30° to the pial surface, with the bevel facing upward to maximise the viral load to the superficial cortical layers. Coordinates, volume, AAV pseudotypes and titres used for each experiment are given in Table 1. Viral constructs were injected at 100 nL/min using a micro-injector (WPI) connected to a Hamilton syringe (5 μL RN; Hamilton Company; #7647-01). The needle was left in situ for 5 min after injection before withdrawing it. The scalp was then sutured, and animals were injected with sterile saline (0.2–0.3 mL, s.c.), and allowed to recover in a heated chamber. Animals were allowed to recover for at least a week before experiments or further surgeries.

#### *In vivo* calcium imaging

Three weeks after viral injection in the upper layer of the visual cortex, NDNF or PV-Cre mice underwent a second surgery to affix a headplate (Model 5, Neurotar Ltd., #NTR000205-02) as described.^50^ In addition to buprenorphine and Metacam, mice were given dexamethasone (2 mg/kg, i.m. PubChem CID 5743, Duphacort Q). A cranial window was performed above the visual cortex (Table 1). The craniotomy was initially outlined with a 2 mm biopsy punch (Kai Medical, Cat #D52421, BP-20F) followed by skull thinning using a right-angled dental drill (NSK) with a diamond burr (Diatech, 862-Flame-Extra Fine 012 Cat #170-0004). Sterilised 2 mm silicon disks (Sylgard 84; 0.31 mm thickness from ref. 51) were secured to a 3 mm glass coverslip with a UV curing optical adhesive (Norland 68, ThorLabs, Cat # NOA68) and placed inside the craniotomy to replace the arachnoid space and avoid formation of air bubbles. Coverslips were glued to the skull using cyanoacrylate adhesive (Vetbond, 3 M™, 7000002814). Posterior to the cranial window, a small burr hole was drilled and a stainless-steel wire was implanted at the cortical surface to record the electrocorticogram (ECoG). The chemoconvulsant was delivered via the same burr hole on the day of experiment. A reference electrode was placed on the contralateral frontal lobe. The headplate and electrode connector were affixed to the skull with opaque dental cement (Super-Bond kit, Sun Medical–K058E #897-4389) and the entire chamber was covered with an opaque silicon-elastomer sealant (Kwik-Cast, WPI Europe, Cat # KWIK-CAST) to protect the cranial window. Animals were allowed to recover for 5 days before habituating to the head-fixed system for increasing periods (10–40 min) over at least 7 days.

#### *In vivo* optogenetic and chemogenetic experiments

During the same surgery to inject AAVs optical adhesive (Norland 68) was applied to the skull above the cortex of interest in preparation for a future cranial window. A headplate (model 5 or 13 Neurotar, Ltd, #NTR000205-02 and #NTR000484-06) was affixed to the skull using opaque dental cement (Super-bond kit as above, Sun Medical) and the site was covered with a layer of opaque silicone elastomer (Kwik-cast as above, WPI Europe). Mice were injected with saline (0.2–0.3 mL, s.c.) and allowed to recover for at least five days before habituation to the head-fixed system. During habituation, water or 30% sucrose was offered to the mice via 1 mL syringe with a blunt end needle.

Three to four weeks later, on the day of the experiment, mice underwent a second surgery to perform a cranial window above the cortex of interest. Mice were anaesthetised and transferred to a stereotaxic frame as described above, and injected with Metacam (2 mg/kg, s.c.) and dexamethasone (2 mg/kg, i.m.). The Kwik-cast and optical adhesive were removed from the skull and a 2 mm craniotomy was then performed as detailed above.

For the optogenetic experiments, the cranial window was not covered but was irrigated with warm sterile saline and covered with silicone elastomer (Kwik-cast). For chemogenetic experiments, sterilised 2 mm silicon disks were placed inside the craniotomy as above. In all cases, animals were injected with 0.2–0.3 mL saline and allowed to recover for several hours before starting recordings.

#### *In vivo* intrahippocampal kainic acid, viral injection and local field potential (LFP) electrode implantation

Mice of both sexes (3–4 months old) underwent two surgeries, the first to induce status epilepticus (and consequently establish chronic epilepsy), and the second to inject AAVs and implant depth electrodes and a subcutaneous wireless transmitter to record LFP signals in the hippocampus. Mice were anaesthetised with isoflurane (5%), and transferred to a stereotaxic frame, and injected with buprenorphine and Metacam as above. They were also injected with bupivacaine (0.02 mL of 0.25% diluted 1:1 in sterile saline, s.c. Marcain, PubChem CID 64737) to the scalp as an incisional block. A burr hole was drilled above the dorsal hippocampus (Table 1, coordinates from bregma, AP: −2.8 mm; ML: 2 mm; DV: −2 mm) and kainic acid (PubChem CID 10255, synthetic, Tocris; 7 mM, 140 nL in males and 7 mM, 80 nL females) was injected into the dentate gyrus at a rate of 200 nL/min (Hamilton syringe, Hamilton Company; 33-gauge flat needle, Esslab Ltd, UK). The needle was retracted 2 min after the end of the injection. The scalp was sutured and mice were administered 0.3 mL saline with glucose (4% w/v, s.c.). Mice were then placed in a heated recovery chamber for 15 min before being returned to their home cage, where they were monitored for 2 h to assess the severity of status epilepticus. Either 2 h after kainic acid injection or after occurrence of three Racine Stage 5 seizures, whichever occurred sooner, mice were given diazepam (10 mg/kg, i.p. PubChem CID 3016, Hameln Pharma) to terminate status epilepticus (SE). Racine stage 5 seizures were defined as loss of righting reflex following rearing and clonus and/or wild jumping.^52^

Two weeks later, mice that had exhibited Racine stage 5 seizures were injected with AAV9-mDlx-FLEX-mCherry or AAV9-mDlx-FLEX-hM3Dq-mCherry and implanted with a wireless transmitter (Open Source Instruments, Single channel, 256 Hz either A3028C-AA-B45-B or A3048S2-AA-C45-D). Briefly, mice were anaesthetised with 5% isoflurane, transferred to a stereotaxic frame and injected with buprenorphine and Metacam as above. AAVs were injected bilaterally in the stratum lacunosum moleculare (SLM) of the hippocampus as above (Table 1). Following viral injection, a small incision was made to the back of the mouse and a wireless transmitter was placed subcutaneously. The leads were tunnelled under the skin through to the skull and a depth electrode was attached to the recording electrode via a metal crimp (Open Source Instruments, SDE-X). A burr hole was drilled (coordinates AP: −3.1 mm; ML: 3.12 mm) and a Teflon-coated stainless steel wire depth electrode was lowered to a DV of −1.5 mm below the pia, 1 mm above the viral injection site in the ventral hippocampus. A reference electrode was placed on the contralateral frontal cortex. The animal was sutured and the electrodes sealed in place with dental cement (Simplex Rapid, Kemdent). Animals were given 0.3 mL saline and placed in a heated recovery chamber, before returning to their home cage where they were allowed to recover for 2–3 weeks before recordings.

### Calcium imaging experiments

#### Acquisition

Imaging data was recorded using an Olympus FV1200MPE multi-photon laser scanning microscope with a 25× immersion objective (XLPLN25XWMP2, Olympus). Head-fixed mice on a floating platform (Neurotar Ltd.) were habituated to the recording setup for a week before recordings. GCaMP6f and mCherry were excited at 910 nm and 800 nm, respectively, using a Ti-Sapphire Chameleon Ultra Pulsed Laser (Coherent). Emission fluorescence was collected using two photomultipliers (PMTs) and filters (PMT 1: 515–560 nm; PMT2: 590–650 nm). Imaging was performed using 254 × 254 μm frame scans (128 × 128 or 256 × 256 pixels) at a frame rate of 15 or 8 Hz, respectively. Resolution and power were kept constant within each experiment and each image acquisition lasted 60–90 s.

To image calcium-dependent fluorescence of NDNF+ and PV+ cells during seizures, mice were anaesthetised with 1.5% isoflurane and transferred to a stereotaxic frame for injection of the chemoconvulsant. Kwik-cast was removed from the headpiece, and pilocarpine (3.5 M, 150 nL, PubChem CID 5909, Tocris, Cat # 0694) was injected via the burr hole located next to the ECoG wire into the visual cortex at a depth of 0.5–0.6 mm and a speed of 100 nL/min using a Hamilton syringe needle (33-gauge, flat end). Mice were then transferred to the head-fixed imaging setup for the recording session. Three to four imaging windows were recorded in each animal approximately 1.5 mm from the chemoconvulsant injection site and ECoG recording wire. The coordinates for each imaging site were recorded from the x,y coordinates of a motorised platform (Scientifica) holding the head-mounting arm. The concomitant ECoG signal was acquired using a Multiclamp 700B amplifier (Molecular Devices), digitised at 10 KHz, bandpass filtered between 0.1 Hz–1 KHz, and recorded using WinEDR (Strathclyde University, UK, RRID:SCR_014270, Strathclyde Electrophysiology Suite). The signal was time-stamped with imaging data by recording a transistor–transistor logic (TTL) signal from the scanning head. ECoG recordings were initiated at the time of chemoconvulsant injection so that the latency to seizures could be monitored. Experiments typically lasted for 40–50 min, and animals were monitored closely for welfare. Mice were offered water and 30% sucrose solution as above. If electrographic seizure activity was observed at the end of the recording session, diazepam (10 mg/kg, i.p.) was administered before returning the mouse to its home cage. In animals where pilocarpine failed to initiate seizures, a second recording session was performed a few days later.

For *in vivo* calcium imaging combined with chemogenetic activation, at least 3 h after surgery, mice were transferred to the head-fixed imaging system and the Kwik-cast was removed from the headpiece. Two to three imaging windows were recorded sequentially in each animal. A baseline was acquired by performing 5 acquisition periods (180 s each) taken 5 min apart. The animal then received either dimethyl sulfoxide (DMSO, PubChem CID 679, Merck-Millipore, Cat #D2650) in saline (1%, i.p.), deschlorclozapine (DCZ, 10 μg/kg in 1% DMSO, PubChem CID 16103, Cambridge Bioscience, Cat # HY-42110) or clozapine (CLZ, 100 μg/kg in 1% DMSO, PubChem CID 135398737, VWR International, Cat # 12059). Image acquisitions resumed 10 min after the injection and were repeated every 5 min for a further 40 min. At the end of the recording session, the silicone elastomer was replaced on the headpiece and the mouse was returned to its home cage. The experiment was repeated over the course of three consecutive days and each day the mice received a different drug or DMSO in saline control. The order of the drug injected for each animal was randomised and the experimenter was blinded to both the virus and the drug injected.

#### Analysis

Image analysis was done using in-house custom MATLAB (2025a, RRID:SCR_001622) scripts. Images from each recording session went through a series of pre-processing steps including frame registration using 2D cross-correlation to correct for small X–Y movements that inevitably occur during seizures and flat-field correction to correct any vignetting. Top-hat filtering using a disk structuring element was done to enhance brightness in objects that had the approximate size and shape of neuronal somata. Regions of interest (ROI) (cell bodies) were then selected for analysis only if they were mCherry positive. Fluorescence signals were extracted from each pixel within the ROI, ignoring those at the soma boundary, to decrease potential contamination from surrounding neuropil. A local background calculated from the area surrounding a cell soma was subtracted from the cell signal, and ΔF/F was calculated using the median cell signal.

Each fluorescence point was aligned to the centre of the TTL pulse in the electrophysiological recordings. Seizures were analysed if they lasted >2 s and spikes were larger than 5 standard deviations (SD) above the noise level. Calcium transients were analysed if they were > 2× SD above background noise. An exponential curve was fitted to the calcium transient to calculate ‘time to plateau’.

### *In vivo* optogenetic experiments

#### Acquisition

Two to 3 h after a craniotomy surgery, pilocarpine was locally injected (3.5 M, 150 nL, 100 nL/min) with a Hamilton syringe and a 34-gauge flat needle (Esslab, Ltd, UK) at the centre of the craniotomy at a depth of 0.5 mm under isoflurane anaesthesia (1.5–2%). The LFP recording system was activated at the start of the injection to create a timestamp. Three minutes after the injection, the needle was retracted and the animal placed in the head-fixed system without further anaesthesia. Typically, within 5 min, the animal would be fully awake. In the meantime, an LFP recording electrode (borosilicate glass pipettes 1.5 mm OD, 0.86 mm ID Warner Instruments, #G150F-4 with a resistance of 2–3 MΩ) filled with saline was positioned at the centre of the craniotomy above the pilocarpine injection site and lowered to 0.3 mm below the pia, and a bare ended optic fibre (ø: 200 μm, NA: 0.22, Multimode 190–1200 nm, Thorlabs Inc., UK) was positioned 1.5–2 mm above the pia. The entire craniotomy and chamber created by the headplate was filled with warm saline and a silver-wire coated with silver chloride was added to the bath for referencing.

The LFP signal was acquired using a Multiclamp 700 B amplifier, filtered between 1 and 300 Hz with a gain of 50 (mV/mV) and digitised at 1 kHz using a NI board and recorded with WinEDR. The LFP signal was passed to a field-programmable gate array (FPGA) CRIO-9076 integrated controller (National Instruments, USA) which triggered the optogenetic laser system in real-time upon seizure detection. A laser source either at 470 or at 570 nm (CNI laser) was used to activate a Chronos or Archaerhodopsin T (ArchT) construct, respectively. The power at the tip of the fibre was between 10 and 15 mW and kept constant throughout the experiment. Ambient light in the room was kept constant and fairly bright to occlude indirect stimulation of the retina. Indeed, preliminary results in dim light conditions suggested that laser stimulation could occasionally entrain activity in the visual cortex in the absence of opsin expression (normalised activity from baseline condition during laser stimulation 142 ± 16%, n = 3 mice).

Optogenetic stimulation started when the LFP signal was exhibiting interictal spikes (about 20–30 min post-injection as previously reported^11^^,^^21^). Each stimulation lasted 10 s and was interspersed by >30 s non-stimulation periods. For ArchT experiments, continuous illumination was applied while various frequencies of pulsed stimulation were used for the Chronos experiment. For Chronos activation, 2, 4, 8, 10, 20, 50, and 100 Hz pulses (duty cycle 50:50) were applied pseudo-randomly throughout the experiment. For tests of interictal spike optogenetic manipulation, laser stimulation was applied in open-loop. To test the effects of optogenetic manipulations on focal seizures, laser stimulation was applied in closed-loop. Briefly, the sentinel spike at the start of seizure onset was detected via a custom Labview threshold-crossing script run on the FPGA, allowing the electrographic seizure to be detected within ∼100 μs. Upon detection, the laser stimulation (either continuous or pulsed) was activated for 10 s with or without a delay. Finally, during closed-loop stimulation, optogenetic stimulation (laser on) was alternated with sham stimulation (detection of the event without laser on).

At the end of the experiment, animals were culled and transcardially perfused with paraformaldehyde (4% PFA, Insight Biotechnology, [CAS 30525-89-4], Cat # sc-281692). The fixed brains were then sliced to 100 μm thickness and analysed to verify expression of the optogenetic actuator and quantify its spread (typically between 2 and 3 mm along the postero–anterior axis, Chronos: mean 2.19 ± 0.12 mm, n = 15 mice; ArchT: mean 2.82 ± 0.08 mm, n = 13 mice). Ectopic expression of viral constructs in pyramidal neurons was estimated by counting the number of fluorescent pyramidal shaped cells within all 100 μm slices containing the viral construct.

#### Analysis

All data were analysed using in-house custom Python scripts. After an initial correction for artefacts, LFP traces were downsampled to 100 Hz and filtered using a Butterworth bandpass filter (1–30 Hz). The onset of interictal discharges was determined by applying a detection threshold to the second derivative of the LFP recording, set at a minimum of 5 standard deviations (SDs) above baseline.

Seizure events were defined according to established criteria.^11^^,^^21^ They must begin with a sentinel spike followed by oscillatory/spiking activity lasting at least 1 s, with the second derivative of electrophysiological trace reaching at least 3 SDs above baseline. The average seizure duration in the control condition across all tested animals was 6.91 ± 0.80 s (n = 19 mice).

The effects of optogenetic manipulations were assessed by comparing the duration of each seizure with photostimulation applied at various delays from seizure onset (test) to that of an immediately preceding seizure (control).^21^ However, if the control seizure was shorter than the delay to photostimulation, the photostimulation trial was excluded from analysis to avoid a bias toward photostimulation-induced prolongation of activity. Furthermore, to guard against confounding effects of slow changes in seizure duration during the experiment, sham trials were interspersed where the laser was not activated but the seizure was detected and measured in the same way. A minimum of two photostimulation trials per condition (e.g. light OFF-light ON, light OFF-sham with different delays or frequency of stimulation) were included per animal. On average, 15 ± 1 photostimulation trials were performed per experiment (median: 15, min: 4, max: 28, n = 19 mice), and 11 ± 1 sham stimulations (median: 12, min: 2, max: 19, n = 15 mice). For experiments in which photostimulation was delayed by >2 s, an average of 6 ± 1 photostimulation trials per experiment (median: 5.5, min: 3, max: 11, n = 8 mice) and 5 ± 1 sham stimulations per experiment (median: 3.5, min: 2, max: 11, n = 8 mice) were analysed. Finally, the mean photostimulation delay for the experiment with a delay >2 s was 3.51 ± 0.33 s for the photostimulation condition and 4.24 ± 0.50 s for the sham condition (n = 8 mice per group, unpaired t-test, p = 0.25).

### Chemogenetic activation of NDNF+ cells in chronic kainate epilepsy model

#### Acquisition

Two weeks after viral injection and LFP transmitter implantation, transmitters were switched on to record at least 1 h of baseline. Mice were then injected with either DCZ (10 μg/kg in 0.1% DMSO, Tocris), i.p., or DMSO (0.1%, Sigma). LFP was recorded for at least 60 min post-injection before switching transmitters off. Two days later, the transmitter was turned on for a baseline recording of 1 h before intraperitoneal injection of either DMSO or DCZ (so that each mouse received an injection of both compounds across the two days). The experimenter was blinded to both the virus and drug injected.

#### Analysis

One hour of LFP recording pre- and post-injection was analysed. LFP recordings were annotated using in-house software (PyECoG2, https://www.pyecog.com/) to classify electrographic activity into 3 types of events;seizures, interictal activity and baseline. Seizures were classified as multiple spiking events that were 2× the standard deviation of the noise and lasted at least 10 s. Two events that occurred within 5 s of each other were counted as one seizure. Baseline was 30 s of recording that contained 6 spikes or fewer. Interictal activity was defined as multiple spike events that lasted <10 s. Periods of discontinuous LFP signal were discarded.

### *Ex vivo* electrophysiology

#### Brain slicing

Male and female NDNF-Cre mice aged 2–4 months injected with AAVs (Table 1) were briefly anaesthetised and given a terminal dose of intraperitoneal sodium pentobarbital (500 mg/kg). Following cardiac perfusion with ice-cold sucrose solution, the brain was removed. Sucrose solution contained (in mM): Sucrose (75, PubChem SID 24899423, Sigma, Cat #S0389), NaCl (87, PubChem SID 329824637, Sigma, Cat #S9888), Glucose (24, PubChem SID 24895335, Sigma, Cat #G8270), NaHCO3 (26, PubChem SID 329824559, Sigma, Cat #S6014), KCl (2.5, PubChem SID 24898635, Sigma, Cat #P5405), MgCl (7, PubChem CID 5360315, Fisher Scientific, Cat # 15656060) NaH2PO4 (1.25, PubChem SID 24899443, Sigma, Cat #S0751) and CaCl2 (0.5, VWR International, Cat # 21114 [CAS 10043-52-4]), oxygenated with carbogen (95% O2/5% CO2).

For cortical slices, sagittal slices were made at 300–400 μm thickness using a vibrating microtome (Leica VT1200S, Leica). For hippocampal slices, 400 μm horizontal sections were cut as described.^53^ All slicing was performed in ice-cold slicing solution bubbled with 95% O_2_/5% CO_2_. Slices were allowed to recover for 30 min in a slicing solution warmed to 35 °C and then allowed to rest at room temperature for 30 min prior to experiments.

Slices used for *ex vivo* seizure models were transferred to an interface chamber filled with storage solution containing: NaCl (119), NaHCO_3_ (26), Glucose (10.92), KCl (2.5), NaH_2_PO_4_ (0.93), CaCl_2_ (2) and MgCl_2_ (1). The chamber was provided with humidified carbogen.

### Local field potentials and *ex vivo* seizure models

#### Acquisition

Slices were suspended on a net within a recording chamber on the stage of an upright microscope. This allowed the recording solution to perfuse both sides of the slice, improving the reliability of initiating seizure-like events. Slices were continuously perfused with a low Mg^2+^ recording artificial cerebro-spinal fluid (aCSF) containing (in mM): NaCl (119), NaHCO_3_ (26), Glucose (22), KCl (3.5) and CaCl_2_ (2.5) at 10 mL/min, heated to 35 °C. For experiments using cortical slices 0.05 mM MgCl_2_ was included in the recording aCSF, whilst for those using hippocampal slices 0.15 mM MgCl_2_ was added. Slices were visualised using an upright microscope (Olympus BX51WI) equipped with differential interference contrast illumination, a water immersion 20× objective (Olympus XLUMPlan FLN, 1.00 NA) and a charge-coupled device (CCD) camera (Ikegami, ICD-47E). Prior to each slice experiment, mCherry fluorescence expression was checked using a 590 nm LED (Thorlabs M590L2) with a mCherry filter cube (Semrock, mCherry-40LP-A-OMF).

LFP recording was performed either from layer 5 of the visual cortex, in a region with mCherry fluorescence in layer 1, or from layer 5 of the entorhinal cortex where mCherry fluorescence was present in the SLM of the hippocampus. LFP was recorded using borosilicate glass pipettes (1.5 mm OD, 0.86 mm ID Warner Instruments, G150F-4), with a resistance of 2–3 MΩ, filled with recording aCSF. LFP signals were acquired with a Multiclamp 700 B amplifier connected to a National Instruments board (NI USB-6341) and a computer running WinWCP (University of Strathclyde, RRID: SCR_014713). Signals were low pass filtered at 1 KHz and digitised at 10 KHz.

A tungsten concentric bipolar electrode (WPI Europe, Cat # TM33CCINS) was used to electrically stimulate layer 5 of the visual cortex or layer 2/3 of the entorhinal cortex to generate seizure-like events (SLEs) from a defined focal region. Electrical stimulation trains (100 Hz for 1 s, 0.1 ms step) of increasing current intensity (20 μA–200 μA) were applied in 20 μA increments via constant current stimulator (Digitimer Ltd, #DS3 689) until a seizure-like event (SLE) was initiated. A SLE was defined as a minimum of 4 consecutive short discharges. The current required to elicit an SLE was defined as the threshold and 110% of this threshold was then used subsequently to elicit SLEs reliably. Slices were allowed to rest for 10 min following the first SLE. Test SLEs were elicited every 5 min with repeated electrical stimulation trains for a total of 12 cycles. Recording aCSF containing the hM3Dq agonist clozapine-N-Oxide (CNO, 10 μM, PubChem CID 135445691, Cambridge Bioscience CAY25780) was applied immediately following the 6th stimulation.

#### Analysis

Average parameters from SLEs generated by stimulations 1–6 formed the ‘baseline response’ and averaged parameters from SLEs at stimulations 10–12 formed the ‘treatment response’. To allow time for diffusion of CNO into the slice, stimulations 7–9 were not analysed. SLEs were analysed using in-house custom Python3 (RRID:SCR_008394) scripts. Briefly, recordings were low pass filtered at 40 Hz and spike detection was performed on 2-min portions of the recording immediately following an electrical stimulation. SLE detection consisted of counting spike events 5× larger than the SD of the baseline noise. The experimenter was blinded to the virus injected throughout the recording and analysis period.

### Whole-cell patch clamp recordings

#### Acquisition

Following a 30-min recovery period at room temperature, slices were transferred to a submerged recording chamber and continuously perfused with aCSF containing (in mM): NaCl (125), NaHCO_3_ (25), D-glucose (25), KCl (2.5), NaHPO_4_ (1.25), CaCl_2_ (2) and MgCl_2_ (1), heated to 35 °C, at 2 mL/min. Slices were held in place using a platinum harp with nylon threads and visualised using an upright microscope as above. mCherry or GFP fluorescence was used to identify NDNF+ interneurons expressing either chemogenetic, or optogenetic actuators as well as control reporters. Borosilicate glass pipettes (1.5 mm OD, 0.86 mm ID Warner Instruments, #G150F-4) with a resistance of 2.5–3.5 MΩ and filled with a solution containing (in mM): K-gluconate (122, PubChem SID 24898248, Sigma, Cat #P1847), KCl (6), HEPES (10, PubChem SID 24895572, Sigma, Cat #H3375), Na-phosphocreatine (10, PubChem SID 24898895, Sigma, Cat #P7936), Mg-ATP (4, PubChem SID 24891422, Sigma, Cat # A9187), Na-GTP (0.4, PubChem SID 57651277, Sigma, Cat # 51120), EGTA (0.2, PubChem SID 24894530, Sigma, Cat #E3889) and MgCl2 (1) were used to record intrinsic and active electrical properties of NDNF+ interneurons. Current clamp recordings were achieved using a Multiclamp 700 B amplifier (Molecular Devices) connected to a National Instruments board (NI USB-6341) and a computer running WinWCP (University of Strathclyde, RRID: SCR_014713) or an in-house LabVIEW (NI) acquisition program. Signals were filtered at 10 kHz and digitised at 50 kHz. Cells that had a series resistance >25 MΩ or a resting membrane potential > −50 mV were not included.

#### For optogenetic depolarisation and hyperpolarisation construct validation

For the characterisation of optogenetic activation of NDNF+ cells, trains of 1 ms-long light stimuli, with 5 pulses at 1 Hz, 10 pulses at 2 Hz, 20 pulses at 5 Hz, 20 pulses at 10 Hz, 20 pulses at 20 Hz, 20 pulses at 50 Hz, 20 pulses at 80 Hz and 20 pulses at 100 Hz, were delivered three times consecutively, with inter-train intervals of 15 s. This was done while maintaining the resting potential at −70 mV in current-clamp mode. For the characterisation of inhibitory post-synaptic potentials (IPSPs) in pyramidal neurons elicited by NDNF+ cell photo-activation, the following protocol was looped twice with a 60 s long inter-stimulus interval: 15 pulses at 5 Hz, 30 pulses at 10 Hz, 60 pulses at 20 Hz, 150 pulses at 50 Hz and 300 pulses at 100 Hz 1 ms-long pulse stimulations. This was done while maintaining the cells at −60 mV in current-clamp mode. Current steps were injected in order to verify the identity of the cell.

#### For chemogenetic construct validation

Action potentials were initiated via a series of depolarising current steps (from 0 to 200 pA increment 25 pA, 1 s long step) from a holding potential of—60 mV.

#### Analysis

Analysis of action potentials was done using the event detection feature in Clampfit (Molecular Devices, v10.7) or custom Python scripts using a threshold detection above −20 mV. For the characterisation of IPSPs, maximum peak hyperpolarisation was measured by subtracting the peak from the baseline of the response traces. The IPSP area was measured by summing the absolute value of each point subtracted by the baseline and dividing by the sampling frequency. The IPSP area was normalised to the peak hyperpolarisation for each trace in order to compare different stimulation frequencies between cells.

### Immunohistochemistry

NDNF-Cre, PV-Cre and SOM-Cre mice expressing either chemogenetic or optogenetic constructs were injected with a lethal dose of sodium pentobarbital (500 mg/kg) and transcardially perfused with cold 4% paraformaldehyde (PFA) in phosphate buffered saline (PBS, PubChem CID 24978514, Sigma, Cat #P4417). Brains were removed, transferred to 4% PFA solution and stored overnight at 4 °C.

#### Immunofluorescence

Using a vibrating microtome (Leica VT1000s), 70–100 μm thick horizontal or coronal brain slices were cut. Free-floating sections were washed 3 × 5 min in 1 × PBS and incubated with a blocking solution (3% goat serum, RRID:AB_3718175, Sigma, Cat #G9023; 0.3–0.5% Triton X-100 Sigma, Cat # × 100, [CAS 9036-19-5], 0.5% bovine serum albumin, BSA, Sigma, Cat # A3311, [CAS 9048-46-8]; in PBS) at room temperature for 1 h. Sections were then incubated with primary antibody in blocking solution (Table 2) at 4 °C overnight on a shaking platform. Primary antibody solutions were then removed, and sections were washed 3 × 10 min with PBS before incubation with a secondary antibody (Table 2) for 3 h at room temperature. Sections were then washed for a further 3 × 5 min in PBS then incubated with DAPI (1:5000, PubChem CID 2954, Invitrogen, Cat #D1306) for 5 min before a further 3 x 5-min washes. Sections were then mounted onto glass slides using a hard-set mounting medium (Fluoroshield, Sigma, Cat #F6182).

**Table 2:** Antibodies.

Antibody	Species	Dilution	Blocking solution	Product code
Anti-parvalbumin	Mouse	1:1000	0.5% Triton X-100 0.5% BSA in PBS	RRID:AB_477329, Sigma P3088
Anti-somatostatin	Rabbit	1:200	0.5% Triton X-100. 0.5% BSA	RRID:AB_10903864, Abcam ab111912
Anti-reelin	Mouse	1:1000	0.2% Triton X-100. 0.5% BSA	RRID:AB_1603148, Abcam ab78540
Anti-neuropeptide Y	Rabbit	1:1000	0.3% Triton X-100. 0.3% BSA	RRID:AB_2307354, Immunostar 22940
Anti-vasoactive intestinal polypeptide	Rabbit	1:500	0.3% Triton X-100. 0.3% BSA	RRID:AB_10730725, Immunostar 20077
Alexafluor488 anti-mouse	Goat	1:1000	PBS	RRID:AB_2534069, Invitrogen A11001
Alexafluor594 anti-rabbit	Goat	1:1000	PBS	RRID:AB_141359, Invitrogen A11012
Alexafluor568 anti-rabbit	Donkey	1:500	PBS	RRID:AB_2534017, Invitrogen A10042
Alexafluor568 anti-mouse	Goat	1:500	PBS	RRID:AB_2534072, Invitrogen A11004

#### Image acquisition

Images were acquired on a Leica Mica or Leica LSM 170 confocal microscope through 20× (0.75 NA) non-immersive objective using laser wavelengths of 405 nm, 488 nm and 594 nm. The laser power and digital gain were adjusted for each staining condition to ensure optimal fluorophore visibility and kept the same within the same immunohistochemical condition.

#### Image analysis

The confocal images were processed using FIJI software. After opening the images, the z-stack projections were collapsed into a single 2D image. To optimise image clarity and accuracy, the brightness/contrast was adjusted, and background noise was subtracted. Neuronal quantification was performed manually using the “cell counter” analysis plugin.

### Sample sizes

Sample sizes were estimated on the basis of pilot data and previous experience using related imaging, optogenetic, and chemogenetic approaches in *ex vivo* and *in vivo* animal models of epilepsy.^10^^,^^11^^,^^21^^,^^54^, ^55^, ^56^, ^57^, ^58^ To obtain a representative sample of population neuronal activity, 20–100 neurons from at least 3 to 5 mice were recorded.^11^^,^^13^^,^^17^^,^^18^ A constraint is the fact that interneurons are sparse compared to principal neurons. For optogenetic experiments according to our previous study,^21^ between 5 and 12 mice are necessary to see an effect size (d) of 0.90–2.40 with a power of 80% and an alpha of 0.05. Using the resource equation approximation method^59^ between 6 and 11 slices per group are optimal for the chemogenetic manipulations *ex vivo* (minimum: 10/k+1, maximum: 20/k+1, number of groups k = 2) and between 4 and 6 animals per group for the *in vivo* study (minimum: 10/kr+1, maximum: 20/kr+1, number of groups k = 2, number of repeated measurements r = 2). Immunostaining was performed on a minimum of 3 slices from at least 2 animals. Calcium imaging was conducted on a minimum of 3 mice per condition, with at least 2 cells imaged per mouse (total >20 cells). For optogenetic experiments, a minimum of 12 mice per condition (Chronos or ArchT) were initially recorded. However, not all animals developed seizures; some exhibited only interictal spikes or continuous epileptiform activity, as described previously,^21^ thereby reducing the number of animals available for seizure analysis (either all pooled or with a delay >2 s). All animals exhibiting interictal spikes were analysed for optogenetic stimulation during interictal spiking activity. For chemogenetic *ex vivo* experiments, between 5 and 8 slices (with a minimum of 1 slice per animal) per condition (mCherry vs. hM3Dq) were recorded and analysed. Finally, for the chronic model of epilepsy, an initial cohort of 13 potentially epileptic mice were injected, implanted and recorded until the end of the experiment. The animals were separated in 2 groups with either mCherry or hM3Dq giving 6 mice with mCherry and 7 with hM3Dq. However, only 3 mice per condition exhibited focal seizures and were subsequently included in the analysis.

### Statistics

All statistical analyses were performed using GraphPad Prism (version 10, RRID:SCR_002798) software or Python3 scripts. In order to visualise effect sizes, mean difference plots for paired and unpaired data were generated using the Data Analysis with Bootstrapped ESTimation package (DABEST,^60^). 95% confidence intervals of the mean difference were calculated by bootstrap resampling with 5000 resamples. The confidence interval is bias-corrected and accelerated to account for the skew but still get the central 95% of the distribution. For two-group unpaired or paired comparisons, normality was not tested as the resampling distribution should approach normality, and two-sided unpaired and paired permutation t-test were instead performed as appropriate. For each test, 5000 reshuffles of the control and test labels were performed. For multi-group comparisons, normality of the data was first tested with the Shapiro–Wilk test, then one-way or two-way ANOVAs were used followed by a post-hoc test when appropriate. Statistical tests, sample and effect sizes, confidence intervals and p values are reported in the figure legends.

### Role of funders

The funders had no role in designing the experiments, collecting and analysing the data, interpreting the results, or writing the manuscript.

## Results

### NDNF-cre allows targeting of layer 1 cells with a profile typical of NGF interneurons

**Fig. 1:**
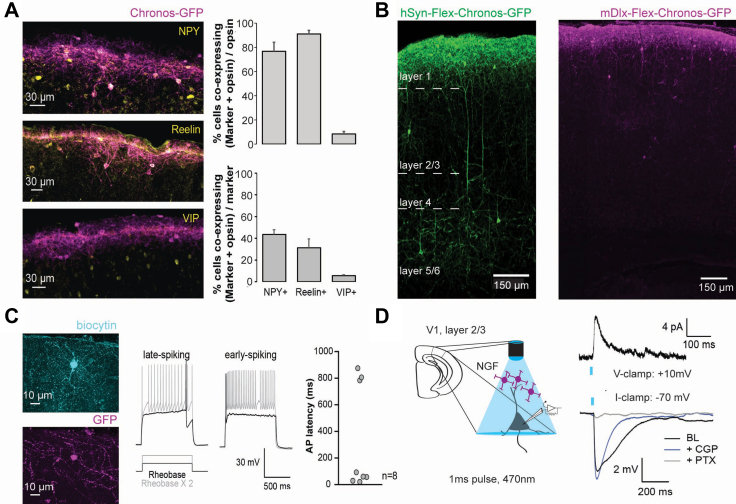
**NDNF-Cre allows layer 1 NGF cells to be targeted.** (**A**) Co-staining of Chronos-GFP (pink) with known molecular markers of NGF neurons, neuropeptide Y (NPY; Top, yellow) and reelin (Middle, yellow) and with a marker not present in NGF neurons, vasoactive intestinal polypeptide (VIP; Bottom, yellow, scale bar: 30 μm). (Right, Top panel) Percentage of Chronos-GFP positive neurons expressing each of the three markers. n = 3 slices, 2 mice. (Right, Bottom panel) Percentage of cells expressing each marker that also express Chronos-GFP. n = 3 slices, 2 mice (**B**) (Left) Example GFP expression (green) in the cortex of an NDNF-Cre mouse injected with an AAV containing a non-specific neuronal promoter, AAV9-hSyn-flex-Chronos-GFP (scale bar:150 μm). Note that some expression is seen within pyramidal shaped neurons in cortical layers 5/6. (Right). Example GFP expression (magenta) in the cortex of an NDNF-Cre mouse injected with an AAV containing an interneuron specific promoter, AAV9-mDlx-flex-Chronos-GFP. Expression is restricted to layer 1 interneurons. (**C**) A patched NDNF+ cell in cortical layer 1 expressing Chronos-GFP (magenta) labelled with biocytin (cyan) via the patch pipette (scale bar: 10 μm). Patched cells showed two characteristic firing patterns, late-spiking and early-spiking. (**D**) Schematic of experimental setup. (Right, Top) Inhibitory post-synaptic current (IPSC) recorded in voltage clamp from a pyramidal neuron in response to activation of layer 1 NDNF+ neurons exhibiting a slow GABA_A_ current. (Right, Bottom) Inhibitory post-synaptic potential (IPSP, black trace; BL–baseline) recorded in current clamp in response to activation of layer 1 NDNF+ neurons. Blocking GABA_B_ receptors with CGP 55845 (navy) abolished the slow component of the IPSP. Addition of picrotoxin abolished the residual IPSP. Error bars correspond to the SEM.

We first sought to confirm that the NDNF-Cre driver line preferentially targets NGF cells using immunohistochemistry and electrophysiological characterisation following AAV-hSyn-FLEX-Chronos-GFP injection. Immunostaining of layer 1 of the visual cortex revealed co-expression of known molecular markers of NGF neurons, including reelin and NPY (Fig. 1A) and little or no VIP co-expression. However, GFP expression was also seen in pyramidal-shaped neurons within deeper layers (Fig. 1B). In order to restrict gene expression to interneurons, we switched hSyn to the interneuron specific enhancer mDlx.^61^ When AAV9-mDlx-FLEX-Chronos-GFP was injected in the superficial cortex of NDNF-Cre mice, GFP expression was almost exclusively seen in layer 1 (Fig. 1B) with only occasional ectopic fluorescent cells (<5 pyramidal shaped cells per spreading area). Layer 1 NDNF+ neurons exhibited either an early-spiking or a late-spiking phenotype when injected with depolarising step current waveforms, as previously reported.^6^^,^^7^^,^^10^^,^^44^ In addition, optogenetic depolarisation of NDNF+ neurons evoked long-lasting IPSPs in pyramidal neurons, mediated by GABA_B_ and GABA_A, slow_ receptors (Fig. 1C and D).

Taken together, the molecular and electrophysiological features of NDNF+ cells described here are consistent with those previously reported, and our intersectional strategy using NDNF marker in combination with an interneuron-specific enhancer, mDlx, allows us to selectively target NDNF+ interneurons in cortical layer 1.

### NDNF+ cell calcium fluorescence during epileptiform activity

**Fig. 2:**
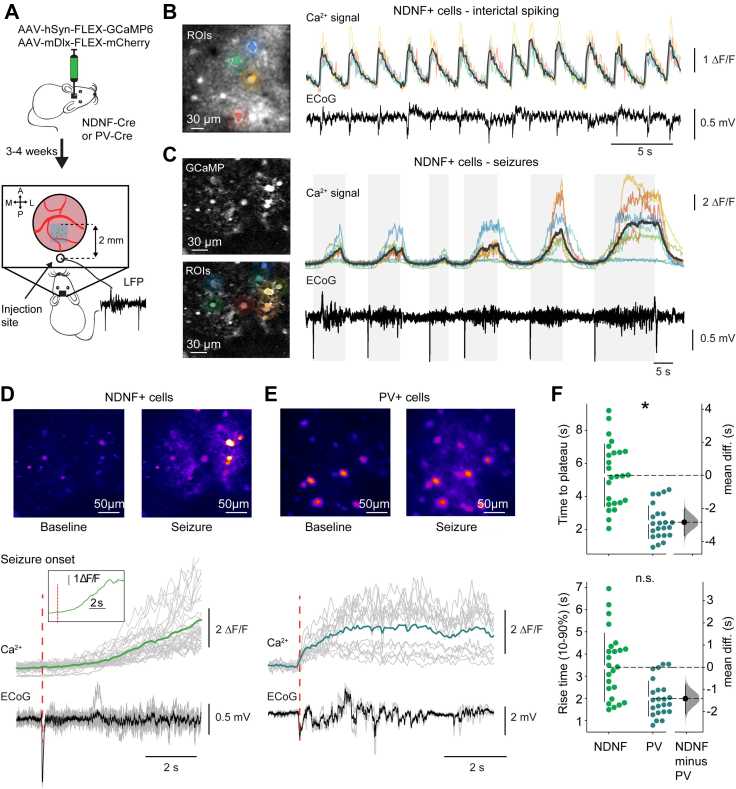
**Delayed recruitment of NDNF+ neurons during seizures.** (**A**) Experimental setup. (**B**) (Left panel) Representative field of view in the cranial window and multicoloured region of interest (ROI) masks on mCherry-positive neurons. (Right panel) Coloured lines depict calcium transients during interictal spikes from individual cells and correspond to neurons in the field of view (Left panel, scale bar: 30 μm). The dark grey lines indicate average calcium transients for all neurons in the field of view. (**C**) (Left panel) Representative field of view (Top) and with ROI masks applied (Bottom, scale bar: 30 μm). (Right panel) Calcium transients (Top) detected in NDNF+ neurons in response to pilocarpine-induced seizures recorded by ECoG (bottom). Coloured lines correspond to calcium transients from individual neurons depicted by coloured masks on image to the left. Dark grey line indicates average calcium fluorescence from all neurons within the field of view. (**D**) (Top panel) Calcium-dependent fluorescence during baseline (Left) and seizure episodes (Right) in an NDNF-Cre mouse (scale bar: 50 μm). (Bottom panel) Average ECoG recording (black line) and calcium transient (dark green line) detected from neurons across multiple seizures within one imaging period (120 s). Individual seizures are shown in grey. (Inset) Average calcium response on an expanded time scale. (**E**) Calcium fluorescence in PV+ interneurons studied as in (D, scale bar: 50 μm). Average calcium transient in PV+ cells shown in blue. (**F**) Average time for the calcium transient to plateau from seizure start in NDNF+ (green) and PV+ (blue) cells (Top panel) and average 10–90% calcium transient rise time (Bottom panel). The time to plateau mean difference is −2.84 s [95%CI, −3.65, −2.04], nested t-test p = 0.023 and the rise time mean difference is −1.42 s [95%CI, −2.11, −0.79], nested t-test p = 0.12; NDNF-Cre n = 26 cells, 5 mice; PV-Cre n = 26 cells, 3 mice. Error bars correspond to the 95% confidence intervals.

Whilst activity of PV+, SOM+, and VIP+ interneurons has been described during hyperexcitable states such as interictal discharges and seizures in *ex vivo* and *in vivo* animal models,^12^^,^^13^^,^^17^, ^18^, ^19^ little is known about the activity of NDNF+ NGF cells in these conditions. We co-injected AAV9-hsyn-FLEX-GCaMP6f and AAV9-mDlx-FLEX-mCherry into the superficial layer of the visual cortex of NDNF-Cre mice in order to record calcium activity in NDNF+ neurons with concurrent electrocorticogram (ECoG) recordings. mCherry fluorescence allowed NDNF+ cells to be identified independently of their calcium activity. On the day of the experiment, pilocarpine was injected in layer 5 in the vicinity of the imaging window (∼1 mm away), to evoke focal epileptiform activity (Fig. 2A). As previously reported,^11^^,^^21^ 20–30 min post-injection, interictal discharges emerged, which then evolved to frequent focal self-terminating seizures (Fig. 2B and C).

Calcium transients were observed in NDNF+ cells during both interictal spiking (Fig. 2B) and seizures (Fig. 2C, percentage of NDNF+ cells responsive to seizures, mean: 76 ± 8.9% median: 80%). We estimated the latency from seizure onset (defined by a sentinel spike) to the maximal calcium transient and compared between NDNF+ cells and PV+ neurons, which are rapidly recruited during seizures.^12^^,^^17^ The recruitment latency of NDNF+ cells was more than twice that of PV+ neurons (Fig. 2D–F, nested t-test, p = 0.023, NDNF-Cre n = 26 cells, 5 mice; PV-Cre n = 26 cells, 3 mice). The rise time tended also to be longer in NDNF+ neurons than in PV+ cells (Fig. 2D–F, nested t-test, p = 0.12), consistent with their delayed recruitment during seizures.

NDNF+ NGF cells are thus recruited relatively slowly during focal seizures.

### Optogenetic hyperpolarisation of NDNF+ cells during epileptiform activity

**Fig. 3:**
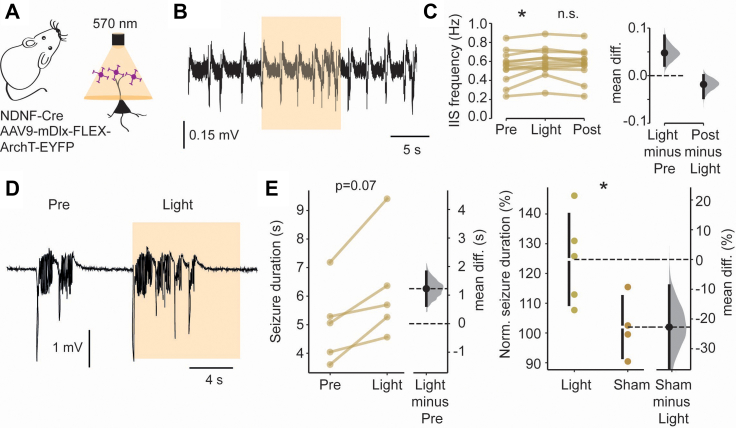
**Optogenetic hyperpolarisation of NDNF+ neurons exacerbates epileptiform activity.** (**A**) Experimental setup. (**B**) Representative ECoG showing interictal spiking evoked by pilocarpine injection. Yellow shading indicates a 10 s long 570 nm illumination of the cranial window. (**C**) Interictal spike (IIS) frequency increased during light exposure (one-way repeated measures ANOVA followed by Šidák post-hoc test; the mean difference between pre-light and light period is 0.05 Hz [95%CI, 0.02, 0.08], p = 0.027 and between light and post-light periods is −0.02 [95%CI, −0.05, 0.001], p = 0.30, effect size (d): 0.12, n = 12 mice). (**D**) Representative seizures before and during NDNF+ cell optogenetic hyperpolarisation. Yellow shading indicates ‘light on’ period. (**E**) Seizure duration was increased during illumination compared to both a preceding seizure (Left panel, the paired mean difference between the duration of the preceding seizure and during NDNF+ cell activation is 1.22 s [95%CI, 0.63, 1.82], p = 0.067, effect size (d): 0.72 two-sided permutation t-test, 5 mice) and sham laser activation (Right panel, the mean difference between the duration of sham and light stimulation is 22.73% [95%CI, 8.2, 38.2], p = 0.0474, effect size (d): 1.74, two-sided permutation t-test, Sham n = 4 mice, Light n = 5 mice). Error bars correspond to the 95% confidence intervals.

We asked if NDNF+ cells restrain or promote network activity during pilocarpine-induced interictal spiking and seizures by activating an inhibitory opsin in awake, head-fixed mice. The hyperpolarising opsin ArchT was expressed in the visual cortex using a mDlx-Cre-dependent viral vector (Fig. 3A), and activated with 570 nm laser illumination delivered via an optic fibre placed above a cranial window. This ensured that the LFP recording and hyperpolarised NDNF+ cells were within the seizure focus. We separately verified that activation of ArchT resulted in hyperpolarisation and suppression of spiking of NDNF+ cells using whole-cell patch clamp recordings in *ex vivo* brain slices (Supplementary Fig. S1).

Optogenetic hyperpolarisation of NDNF+ cells *in vivo* resulted in a small but significant increase in the frequency of interictal spikes (Fig. 3B and C, repeated measures ANOVA followed by Šidák post-hoc test, pre-light vs. light p = 0.027; light vs. post-light p = 0.30, n = 12 mice). This contrasts with the effect of optogenetic hyperpolarisation of PV+ cells, which had very little effect on interictal spiking activity (Supplementary Fig. S2).

To investigate the effect of optogenetic hyperpolarisation of NDNF+ cells during focal seizures, we triggered the laser upon detection of a sentinel spike in real-time. Consistent with the effect on interictal discharges, hyperpolarising NDNF+ cells resulted in an increase in seizure duration (Fig. 3D–E), as determined by comparing either to the immediately preceding seizure where laser illumination was not delivered, or to the effect of sham activation of the opsin where the seizure was detected but the laser was not activated (two-sided permutation t-test, pre-light vs. light, p = 0.067, n = 5 mice; sham vs. light, p = 0.047, sham n = 4 mice and light n = 5 mice). Overall, optogenetic NDNF+ cell hyperpolarisation prolonged the seizure duration by ∼20%.

Taken together, these results suggest that NDNF+ neurons locally restrain pathological activity during both interictal discharges and seizure activity.

### Optogenetic depolarisation of NDNF+ cells during epileptiform activity

**Fig. 4:**
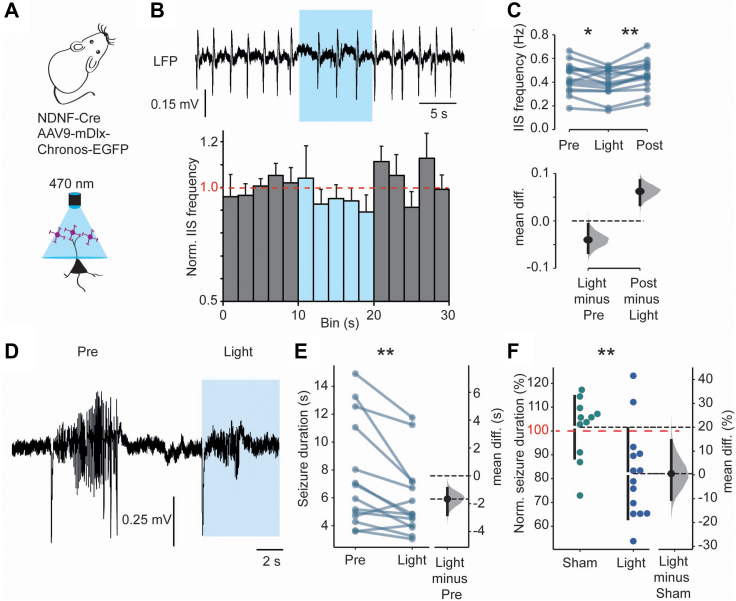
**Optogenetic depolarisation of NDNF+ interneurons consistently reduces epileptiform activity.** (**A**) Experimental setup. (**B**) Representative ECoG recording of interictal spiking following pilocarpine injection. Blue shading indicates 470 nm laser illumination via the cranial window (Error bars correspond to the SEM). (**C**) Interictal spike (IIS) frequency decreased during activation of Chronos (Light) compared to a preceding or following 10 s period (one-way repeated measures ANOVA followed by Šidák post-hoc test; the mean difference between pre-light and light period is −0.04 Hz ([95%CI, −0.07, −0.01], p = 0.046; between light and post-light periods, the mean difference is 0.06 Hz [95%CI, 0.03, 0.09], p = 0.001; effect size (d): 0.21, n = 15 mice). (**D, E**) Seizure duration was decreased during Chronos activation compared to a preceding seizure (the mean difference between the duration of the preceding seizure and during NDNF+ cell activation is −1.66 s [95 CI, −2.8, −0.9], p = 0.003, effect size (d): 0.72, 0.49, two-sided permutation t-test, n = 14 mice) or sham light activation (**F**, the mean difference between the duration of sham and light stimulation is −19.5% [95%CI, −30.4, −5.50], p = 0.009, effect size (d): 1.18, two-sided permutation t-test Sham n = 11 mice, Light n = 14 mice). Error bars correspond to the 95% confidence intervals.

Since inhibiting NDNF+ neurons promoted epileptiform activity, we hypothesised that depolarising NDNF+ cells would have the opposite effect. To test this prediction, we used the same experimental setup but instead expressed the depolarising opsin Chronos in NDNF+ cells (Fig. 4A). We used the same FGPA system to detect the seizure onset and delivered 470 nm laser illumination via the optic fibre to activate Chronos.

We first evaluated several stimulus parameters to optimise the ability to activate NDNF+ cells optogenetically *ex vivo*. We confirmed that trains of 1 ms pulses elicited action potentials in NDNF+ cells using whole-cell patch clamp recordings. Most of the patched cells followed light pulses 1:1 up to 80–100 Hz (Supplementary Fig. S3). However, NGF cells are known to exhibit synaptic depression.^39^^,^^44^^,^^62^ We therefore looked for a stimulating protocol that minimised depression of GABAergic synapses made by NDNF+ cells. Unexpectedly, when a 30 s gap was inserted between 2 stimuli, there was no detectable synaptic depression, and only when the gap was 10 s or less was depression observed. We also investigated whether high frequency stimulation sustained for several seconds resulted in strong depression. Whilst pulse trains elicited synaptic depression, the charge transfer was the biggest for 20 pulses delivered at 20–100 Hz (Supplementary Fig. S3). We thus used a refractory period of a minimum of 30 s between 2 stimulus trains to avoid depression of NDNF+ cell output. We also pseudo-randomly varied the stimulation frequency between 2, 4, 8, 10, 20, 50, and 100 Hz.

Overall, *in vivo* activation of NDNF+ cells using Chronos resulted in a consistent reduction in the number of interictal spikes (Fig. 4B and C) compared to the preceding 10 s period (RM ANOVA followed by Šidák post-hoc test, pre-light vs. light, p = 0.046, n = 15 mice). The number of interictal spikes then returned to baseline levels in the 10 s period following cessation of light exposure (light vs. post-light, p = 0.001). The effect was maximal at a stimulation frequency of 50–100 Hz (Supplementary Fig. S4). The effect of activating NDNF+ cells differed from that of activating PV+ or SOM+ cells because the latter resulted in an initial suppression of interictal spikes which faded and was quickly followed, especially for PV+ cells, by a large overshoot of activity upon terminating optogenetic depolarisation (Supplementary Fig. S5). In contrast, NDNF+ cell stimulation continuously suppresses interictal spiking and only a small but significant rebound was observed after the light was switched off.

When optogenetic depolarisation of NDNF+ cells was triggered by the sentinel spike, seizure duration was reduced by ∼20% when compared to the preceding seizure, whilst sham stimulation had no effect (Fig. 4D–F, two-sided permutation t-test, pre-light vs. light, p = 0.003, n = 14 mice; sham vs. light, p = 0.009, sham n = 11 mice and light n = 14 mice). Consistent with the effect on interictal spiking activity, 50–100 Hz stimulation yielded the strongest effect (Supplementary Fig. S4).

Taken together, the optogenetic manipulations of NDNF+ cells had bidirectional effects on epileptiform activity in the visual cortex, consistent with a role in attenuating runaway excitation.

### GABA_B_-receptor mediated persistent inhibition by NDNF+ cells during seizures

We previously showed that optogenetic activation of PV+ interneurons was anti-epileptic if delivered immediately at seizure onset but was paradoxically pro-epileptic if delayed by more than 2 s from seizure onset. This pro-epileptic effect was abolished when the potassium, chloride co-transporter 2 (KCC2) was overexpressed in pyramidal neurons, suggesting that the somatic transmembrane chloride gradient rapidly collapses during seizures, compromising the inhibitory effect of PV+ cells.^21^ In the case of SOM+ interneurons, their anti-seizure effect also faded rapidly when optogenetic depolarisation was delayed, although conversion to a pro-seizure effect was not observed. We speculated that, since NDNF+ neurons and NGF cells exert their inhibitory effects via dendritic GABA_A, slow_ and GABA_B_ receptors, their ability to inhibit pyramidal neurons may be less labile during seizures.

**Fig. 5:**
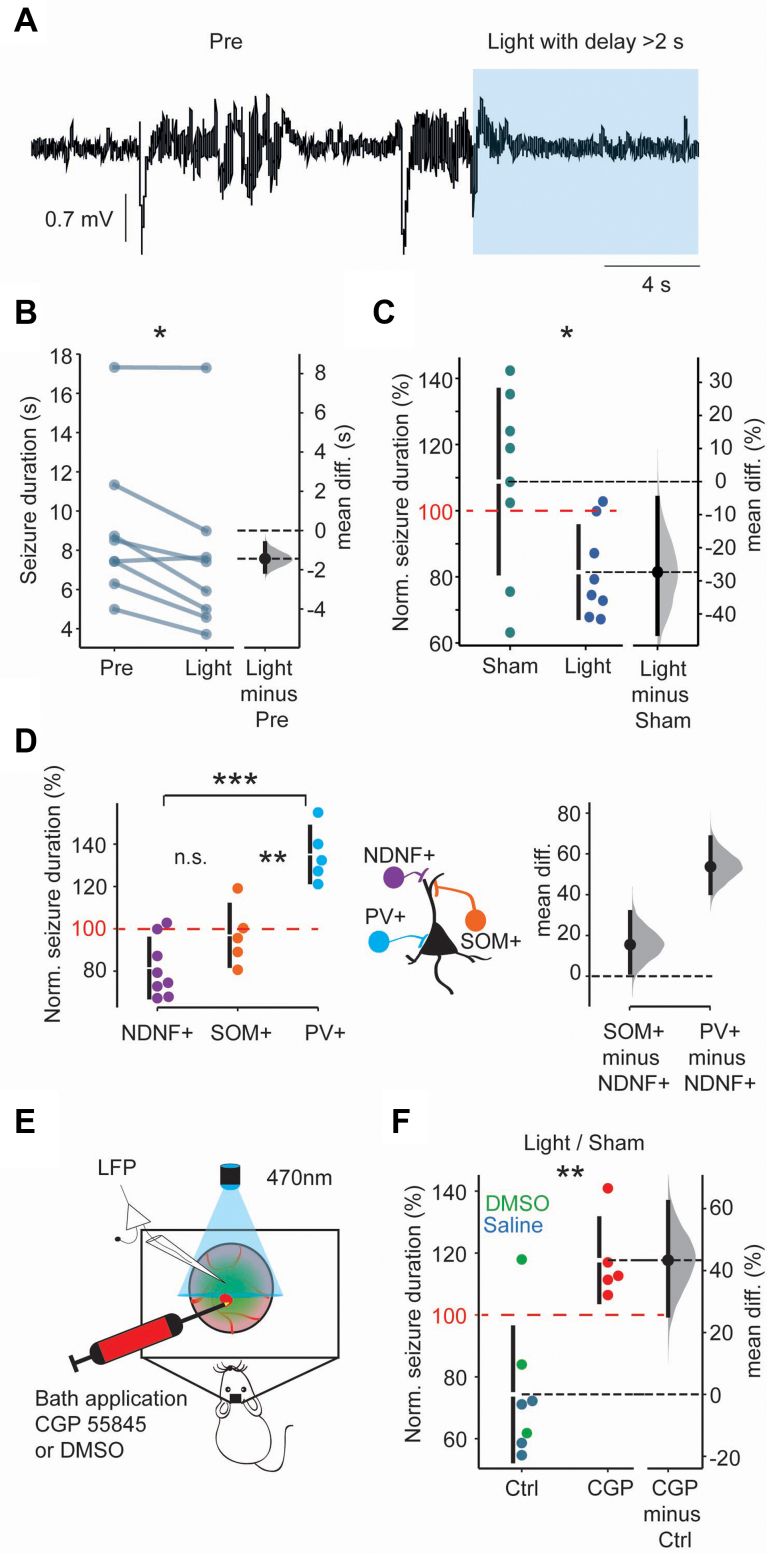
**The anti-seizure effect of NDNF+ cells persists during seizures and depends on GABAB receptor activation.** (**A**) Representative seizures recorded by ECoG from an NDNF-cre mouse. Blue shading indicates 470 nm laser illumination to activate NDNF+ interneurons. (**B**) Seizure duration was significantly decreased by NDNF+ depolarisation even when delayed by >2 s from seizure onset (the mean difference between the duration of the preceding seizure and during NDNF+ cell activation is −1.44 s [95%CI, −2.09, −0.65], p = 0.026, effect size (d): 0.35, two-sided permutation t-test, n = 8 mice). (**C**) Normalised seizure duration was decreased during light exposure compared to sham light exposure (the mean difference between the duration of sham and light stimulation is −27.4% [95%CI, −46.0, −4.95], p = 0.030, effect size (d): 1.25, two-sided permutation t-test, Sham n = 8 mice, Light n = 8 mice). (**D**) Normalised seizure duration following delayed optogenetic activation (>2 s after seizure onset) of NDNF+, SOM+ or PV+ neurons. Delayed activation of NDNF+ neurons decreased seizure duration compared to both SOM+ and PV+ neurons (one-way repeated measures ANOVA followed by Tukey post-hoc test; the mean difference of normalised duration between NDNF+ and SOM+ cell population is 15.49% [95%CI, 1.80, 31,39], p = 0.15, and between NDNF+ and PV+ cells is 53.70% [95%CI, 40.83, 68.12], p < 0.001, effect size (d): 0.85, NDNF+ n = 8 mice, SOM+ n = 5 mice, PV+ n = 5 mice; SOM+, and PV+ cell data from Magloire et al., 2019). (**E**) Experimental setup. Either the GABA_B_ receptor blocker CGP 55845 (10 μM) or DMSO (0.1% in saline) control was applied topically via the cranial window. (**F**) Activation of NDNF+ neurons increased seizure duration in the presence of CGP 55845 (the mean difference between the control and CGP is 43.34% [95%CI, 25.5, 62.1], p = 0.009, effect size (d): 2.40, two-sided permutation t-test, Control n = 7 mice, CGP n = 5 mice). Error bars correspond to the 95% confidence intervals.

To test this hypothesis, we looked at the effect of optogenetic depolarisation of NDNF+ cells delayed by >2 s from seizure onset, as we did previously for PV+ and SOM+ interneurons.^21^ A robust ∼30% decrease in seizure duration was still obtained under these conditions (Fig. 5A–C, two-sided permutation t-test, pre-light vs. light, p = 0.026, n = 8 mice). Seizure duration did not change with sham optogenetic stimulation (Fig. 5C, sham vs. light, p = 0.03, sham n = 8 mice and light n = 8 mice).

NDNF+ interneurons thus retain their inhibitory action during seizure activity, in contrast to PV+ and SOM+ interneurons (Fig. 5D, delay > 2 s, data from ref.^21^; RM ANOVA followed by Tukey post-hoc test, NDNF+ vs. SOM+, p = 0.15, NDNF+ vs. PV+, p < 0.001, NDNF+ n = 8 mice, SOM+ n = 5 mice, PV+ n = 5 mice).

To assess whether this persistent anti-seizure effect of NDNF cells was mediated by GABA_B_ receptors, we repeated the experiments whilst applying either the GABA_B_ receptor antagonist CGP55845 (CGP, 10 μM in 0.1% DMSO) or DMSO (0.1% in saline) or saline as control (Fig. 5E). To improve penetration of the drug, the dura was nicked at ∼5–6 sites. CGP consistently abolished the inhibitory action of NDNF+ neurons: activating NDNF+ cells in the presence of CGP resulted in an increase in seizure duration compared to the preceding seizure (Fig. 5F). Activation of NDNF+ neurons in the presence of DMSO continued to result in reduced seizure duration (two-sided permutation t-test, control vs. CGP, p = 0.009, control n = 7 mice, CGP n = 5 mice).

Taken together these results confirm that the inhibitory effect of NDNF+ cells on the local circuit persists even during excessive network activity such as ictal discharges, in contrast to PV+ and SOM+ cells, which represent about 70% of all interneurons.^5^ This persistent inhibition is principally mediated by GABA_B_ receptors which act independently of the postsynaptic chloride reversal potential.

### Chemogenetic activation of NDNF+ cells is anti-epileptic in cortical and hippocampal recurrent seizure models

Optogenetics remains a vital tool to study the role of specific cells, but the requirement for an optic fibre and light source limits its translational potential. We therefore switched to a chemogenetic approach to activate NDNF+ neurons and interrogate its efficacy in preventing seizures. Chemogenetics not only permits titratable activation of neurons in response to a specific agonist but could also be used as an “as needed” therapy, for instance to prevent impending status epilepticus.^55^ We assessed the effect of chemogenetic NDNF+ neuron activation on seizure burden using both *ex vivo* cortical and hippocampal slice models of seizures and an *in vivo* model of chronic temporal lobe epilepsy.

First, we verified that the excitatory Designer Receptor Exclusively Activated by Designer Drugs (DREADD) hM3Dq can activate NDNF+ cells *ex vivo*. AAV9-mDlx-FLEX-hM3Dq-mCherry, or an mCherry control AAV, was injected into cortical layer 1 of NDNF-Cre mice, and after preparing acute brain slices whole-cell patch clamp was used to record action potential firing before and after the addition of the DREADD agonist clozapine-N-oxide (CNO, 10 μM). The resting input resistance and action potential firing were increased in neurons expressing hM3Dq-mCherry after the addition of CNO to the aCSF (Supplementary Fig. S6).

**Fig. 6:**
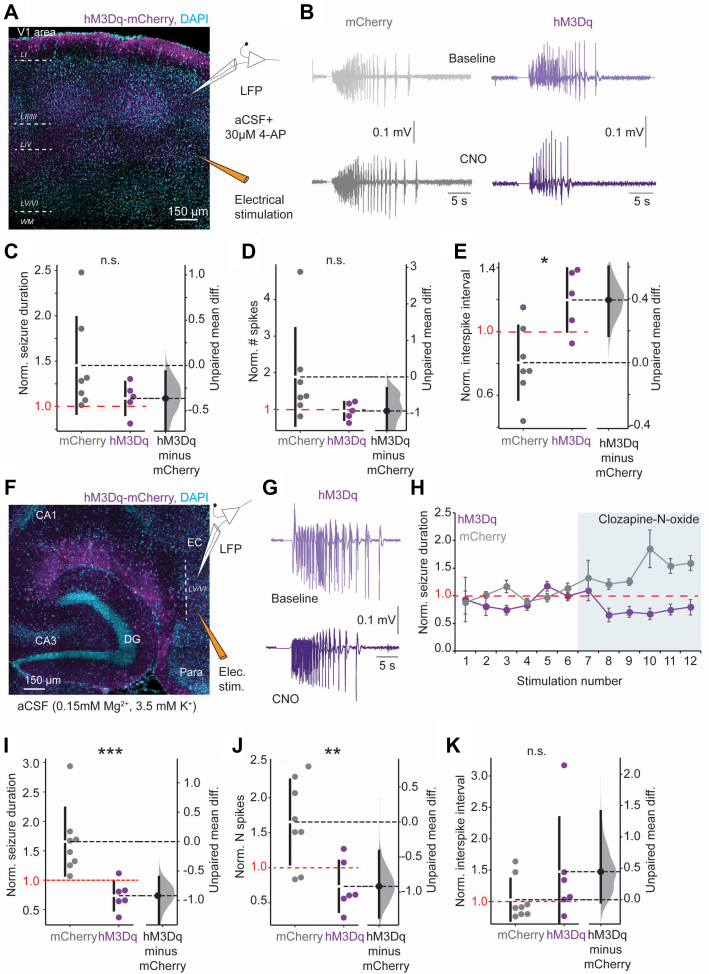
**Chemogenetic activation of NDNF+ cells reduces seizure phenotype in an *ex vivo* cortical and hippocampal models.** (**A**) Cortical slice seizure model setup (scale bar: 150 μm). (**B**) Representative LFP traces of seizure-like events (SLEs) evoked by electrical stimulation from slices expressing either hM3Dq-mCherry (purple), or mCherry only (grey) in NDNF+ neurons in baseline (Top panel) or following addition of 10 μM CNO (Bottom panel). (**C**) Seizure duration following 10 μM CNO application normalised to baseline (the mean difference between mCherry and hM3Dq groups is −0.36 [95%CI, −0.89, −0.06], p = 0.19, effect size (d): 0.91, two-sided permutation t-test, mCherry n = 7 slices; hM3Dq n = 5 slices). (**D**) Normalised number of spikes per SLE baseline (the mean difference between mCherry and hM3Dq groups is −0.93 [95%CI, −2.44, −0.31], p = 0.09, effect size (d): 0.97, two-sided permutation t-test, mCherry n = 7 slices; hM3Dq n = 5 slices). (**E**) Average normalised interspike interval per SLE after CNO application baseline (the mean difference between mCherry and hM3Dq groups is 0.39 [95%CI, 0.16, 0.60], p = 0.01, effect size (d): 1.82, two-sided permutation t-test, mCherry n = 7 slices; hM3Dq n = 5 slices). (**F**) Hippocampal-entorhinal cortical slice seizure model (scale bar: 150 μm). (**G**) Representative LFP recordings of stimulus-evoked SLEs from slices expressing hM3Dq before (top panel) and after (bottom panel) application of 10 μM CNO. (**H**) Duration of SLEs normalised to baseline (before CNO) in slices expressing mCherry only (grey; n = 8 slices/8 mice) or hM3Dq-mCherry (purple; n = 6 slices/6 mice). Error bars correspond to the SEM. (**I**) Normalised seizure duration after CNO was decreased in hM3Dq expressing slices baseline (the mean difference between mCherry and hM3Dq groups is −0.92 [95%CI, −1.49, −0.60], p < 0.001, effect size (d): 2.07, two-sided permutation t-test, mCherry n = 8 slices/8 mice; hM3Dq n = 6 slices/6 mice). (**J**) Number of spikes per SLE was decreased (the mean difference between mCherry and hM3Dq is −0.92 [95%CI, −1.36, −0.41], p = 0.006, effect size (d): 1.82, two-sided permutation t-test mCherry n = 8 slices/8 mice; hM3Dq n = 6 slices/6 mice) and (**K**) interspike interval was increased in hM3Dq slices with CNO (the mean difference between mCherry and hM3Dq is 0.44 [95%CI, −0.04, 1.41], p = 0.19, effect size (d): 0.67, two-sided permutation t-test mCherry n = 8 slices/8 mice; hM3Dq n = 6 slices/6 mice). Error bars correspond to the 95% confidence intervals.

We next asked whether chemogenetic activation of layer 1 NDNF+ neurons reduces the duration of seizure-like events (SLEs) in a cortical brain slice model (Fig. 6A). Circuit excitability was increased by including a low concentration of the voltage-gated potassium channel blocker 4-aminopyridine (4-AP, 30 μM) in the aCSF, and SLEs were triggered by focal electrical stimulation in layer 5 of the cortex, while recording in an area of mCherry expression (Fig. 6A). We observed a non-significant reduction in seizure duration and in the number of spikes within a SLE (two-sided permutation t-test seizure duration mCherry vs. hM3Dq, p = 0.19, number o spikes, p = 0.09, mCherry n = 7 slices/7 mice, hM3Dq n = 5 slices/5 mice), and a significant increase in interspike interval (p = 0.01), compared to slices expressing the mCherry alone, when CNO was added to the aCSF (Fig. 6B–E).

GABA_B_ receptor activation by baclofen is reported to be more potent in preventing epileptic activity in the hippocampus compared to neocortex.^63^ Because NDNF+ NGF cell activation acts in large part via GABA_B_ receptors, we speculated that a hippocampal seizure model would be more suited to test the efficacy of driving them chemogenetically. We previously used NDNF-Cre mice to target NDNF+ NGF cells in the stratum lacunosum moleculare (SLM, ref. 10, Fig. 6F) in the hippocampus, and found a similar electrophysiological profile as in the visual cortex. In addition, NDNF+ cells in the SLM strongly expressed the NGF cell marker reelin (Supplementary Fig. S7). Our intersectional approach therefore also works to target NDNF + cells in the hippocampus. To test the impact of activating NDNF+ cells on hippocampal SLEs, we used low Mg^2+^ (0.15 mM) aCSF to increase neuronal excitability without initiating spontaneous seizures, and triggered SLEs with electrical stimulation of the entorhinal cortex. Following addition of CNO, the duration of SLEs and number of spikes within each SLE were decreased in slices expressing hM3Dq-mCherry, compared to slices expressing mCherry alone, which showed a slow increase (Fig. 6G–K, two-sided permutation t-test seizure duration mCherry vs. hM3Dq, p < 0.001, number of spikes, p = 0.006 mCherry n = 8 slices/8 mice, hM3Dq n = 6 slices/6 mice). Chemogenetic activation of NDNF+ cells thus has a mild anti-seizure effect in the neocortical *ex vivo* models, whilst their activation in the hippocampus has a much stronger impact on epileptiform activity.

**Fig. 7:**
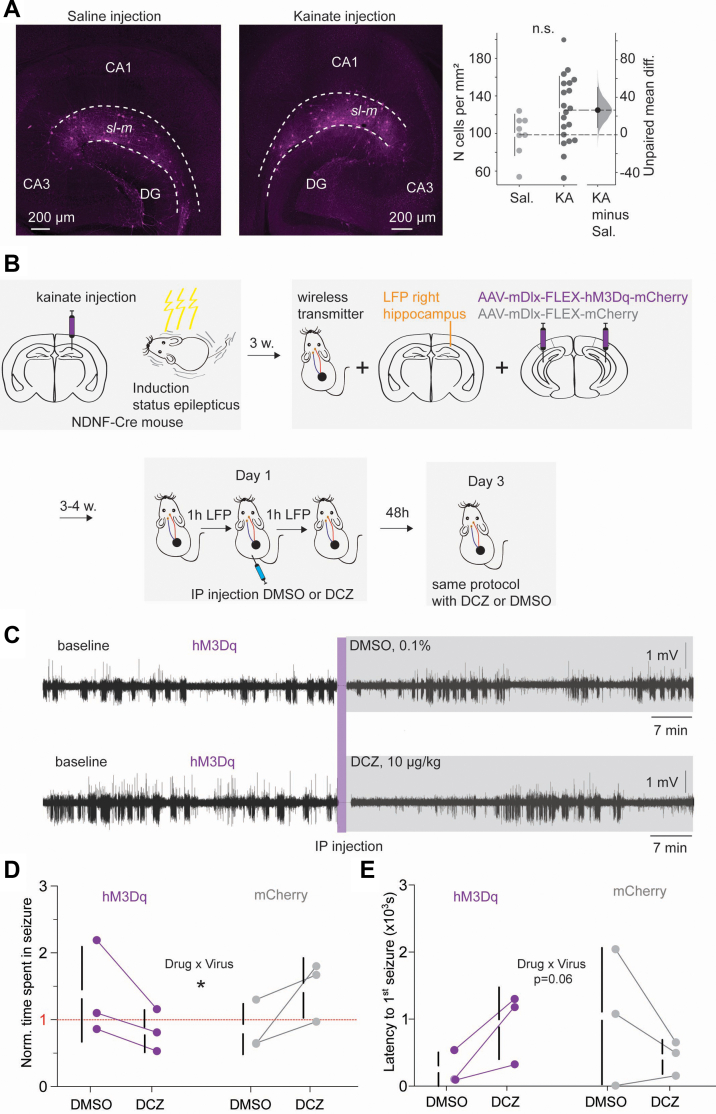
**Chemogenetic activation of NDNF+ cells reduces seizure burden in an *in vivo* chronic model of temporal lobe epilepsy.** (**A**) mCherry expression in NDNF+ neurons within the stratum lacunosum moleculare (sl-m) of the hippocampus from mice injected with either intrahippocampal saline (control) or intrahippocampal kainate (epileptic), scale bar: 200 μm. DG = dentate gyrus. The number of mCherry-positive cells per area was unchanged in epileptic animals (the mean difference between Saline and Kainate is 26,3% [95%CI, 7.8, 50.5], p = 0.072, effect size (d): 0.88, two-sided permutation t-test, Saline n = 8 slices/3 mice; Kainate n = 20 slices/3 mice). (**B**) Experimental setup. (**C**) Representative LFP traces from hippocampal depth electrodes from a mouse expressing hM3Dq in NDNF+ neurons before and after intraperitoneal (i.p) injection of either DMSO (0.1% in saline) control or DCZ (10 μg/kg). Vertical purple line represents time of injection and grey shading indicates post-injection ECoG recording. (**D**) Time spent in seizure was significantly decreased in mice expressing hM3Dq following injection of DCZ (two-way ANOVA, interaction p = 0.033, n = 3 mice per group). (**E**) Latency to 1st seizure post-injection was increased in mice expressing hM3Dq following injection of DCZ (two-way ANOVA, interaction p = 0.062, n = 3 mice per group). Error bars correspond to the 95% confidence intervals.

Although NDNF+ cells are a potential cellular target for novel antiepileptic therapies, loss of interneurons in the hippocampus has been documented in temporal lobe epilepsy (TLE).^64^, ^65^, ^66^, ^67^, ^68^, ^69^ Of the different populations, PV+ interneurons are the most vulnerable, although SOM+ cells are also affected (Supplementary Fig. S8). If NDNF+ NGF interneurons do not survive in chronic epilepsy, this may limit the effectiveness of a chemogenetic therapy targeting this cell type. We therefore asked whether NDNF+ cells are lost in a model of TLE evoked by intra-hippocampal injection of the chemoconvulsant kainate, which triggers a period of status epilepticus followed by frequent spontaneous focal and generalised seizures. The experiments were performed in mice where NDNF+ cells were labelled with mCherry as above. We measured the density of NDNF+ neurons in epileptic animals compared to intrahippocampal-saline injected control mice. We observed no reduction in the number of mCherry + neurons (Fig. 7A, two-sided permutation t-test, p = 0.072, saline n = 8 slices/3 mice; kainate n = 20 slices/3 mice) suggesting that NDNF+ NGF cells are relatively resistant to seizure-related damage in TLE.

In a separate group of awake, head-fixed animals expressing both hM3Dq and GCaMP6f in cortical NDNF+ cells, we verified that the DREADD agonists clozapine (CLZ) and deschlorclozapine (DCZ) which activate hM3Dq *in vivo* directly, unlike CNO which undergoes back-conversion to clozapine,^70^ increase the firing frequency of NDNF+ cells (Supplementary Fig. S9).

Finally, we asked whether chemogenetic activation of hippocampal NDNF+ cells could prevent spontaneous focal seizures in the TLE model. Three weeks following status epilepticus induction, we implanted epileptic animals with wireless transmitters connected to a hippocampal depth electrode to record focal epileptic activity, and injected either hM3Dq-mCherry or an mCherry-only virus bilaterally in the SLM of the hippocampus (Fig. 7B). Two weeks post-injection, the hippocampal LFP was recorded for 1 h (baseline) and then again for an hour post-treatment with either DCZ (10 μg/kg in 0.1% DMSO, i.p.) or DMSO (0.1%) control. Two days later, the same recording was repeated with DMSO or DCZ switched, depending on what the mice received on the first day. The drug allocation was randomised and experimenters were blinded to both the viruses and drugs during the entire experiment and analysis. DCZ injection led to a decrease in seizure burden, defined as the time spent in seizure state, in mice expressing hM3Dq but not mCherry alone, compared to injection of DMSO (Fig. 7C and D), with a significant interaction between virus and substance injected (two-way ANOVA interaction p = 0.033, n = 3 mice per group, Fig. 7D). The latency to the first seizure after drug injection showed a non-significant trend to increase (two-way ANOVA interaction p = 0.062, n = 3 mice per group, Fig. 7E).

Our preliminary experiment on activating NDNF+ interneurons chemogenetically thus suggests an anti-epileptic effect, identifying this population as a candidate cell target for diseases associated with circuit hyperactivity.

## Discussion

Using an intersectional interneuron-specific approach with an NDNF-Cre mouse line, we characterised the role of NDNF+ NGF cells in regulating cortical microcircuit excitability, both in extreme physiological and in pathological conditions. Through calcium imaging and optogenetic manipulation of NDNF+ cells, we demonstrated their substantial contribution to inhibitory restraint even several seconds after seizure onset, a mechanism mainly mediated by GABA_B_ receptor activation. Finally, by enhancing NDNF + cell intrinsic excitability using chemogenetics, we showed that this interneuron class continues to inhibit the cortical circuitry even in pathological epileptic conditions.

We found that approximately 70% of NDNF+ cells express NPY, and 80% reelin. This compares with previous studies that report 30–70% of NDNF+ neurons expressing NPY, depending on the method used (e.g., NPY-GFP mouse line^6^^,^^7^^,^^71^). In addition, a subset of NDNF+ cells exhibited a “late-spiking” (LS) phenotype, a hallmark of NGF cells^9^ whilst other cells displayed an “early-spiking” (ES) pattern. This dual spiking phenotype is consistent with other studies.^6^, ^7^, ^8^^,^^44^^,^^71^ Indeed, Schuman et al. (2019) identified two types of NDNF-expressing cells, NGF and canopy cells, based on their co-expression of NPY, spiking patterns, connectivity profiles, and GABA signalling.^7^ Despite the proposed distinction between these two cell types, their relative proportions within the NDNF+ population remain ambiguous. Our findings along with others, indicate that NPY and reelin are expressed in over 60% of all NDNF+ interneurons (80% for reelin;^6^^,^^44^^,^^71^), contrasting with the 30% reported by Schuman et al. (2019).^7^ Furthermore, Hartung et al. (2024), using unsupervised classification of NDNF+ neurons based on firing patterns and intrinsic properties, found no correspondence between NPY co-expression and the two electrophysiological cell types (ES vs. LS). They suggested that this discrepancy might be due to age-dependent NPY expression, with juvenile animals expressing less NPY.^44^ Although the precise proportion of NGF cells targeted by the NDNF-Cre mouse line remains unclear, canopy cells exhibit a lower connectivity rate with pyramidal neurons (18%) compared to NGF cells (72%), and generate smaller IPSCs that are mediated entirely by GABA_A_ receptors,^7^ suggesting a minor role in inhibiting the cortical column. The inhibitory output of the NDNF+ cell population in our study clearly evokes a powerful long-lasting inhibition mediated by both GABA_A, slow_ and GABA_B_ receptor activation, a signature unique to NGF cells.^6^^,^^44^^,^^71^ Thus, whilst the NDNF+ cell population can be divided into at least two subtypes, it predominantly exhibits an inhibitory profile characteristic of NGF interneurons.

NDNF+ neurons are active during both interictal spikes and focal seizures originating approximately 1 mm away,^11^^,^^72^ suggesting that, like PV+ and SOM+ cells, they can be recruited by local hyperactivity.^12^^,^^13^^,^^17^, ^18^, ^19^ However, their recruitment during focal seizures is significantly slower than that of PV+ cells. Several hypotheses can explain this slow-onset calcium rise in NDNF+ interneurons. Firstly, given that NDNF+ cells are located in layer 1 and receive afferent inputs from distal cortical and subcortical areas,^6^^,^^71^ their gradual activation might result from the progressive recruitment of adjacent cortical territories as the seizure propagates. This mechanism alone is, however, unlikely to fully account for their slow-onset recruitment, as they are also active during local interictal events. A subset of NDNF+ NGF cells exhibit late-spiking activity,^6^^,^^7^^,^^9^^,^^10^^,^^44^^,^^71^ which likely contributes to their delayed recruitment by slowly integrating changes in their membrane potential or incoming activity across hundreds of milliseconds to seconds. Secondly, NDNF+ cells potentially handle intracellular calcium differently than do PV+ cells. This could result in slower calcium entry and/or binding to GCaMP, thereby prolonging the rise time of somatic calcium signals independently of spiking activity. It is however unlikely that differences in intracellular calcium buffering account for this effect, since PV+ cells, despite their high expression of the calcium-binding protein parvalbumin, exhibit faster calcium signal rises. Lastly, NDNF+ NGF cells possess very short dendrites^8^^,^^9^^,^^35^ and demonstrate N-methyl-D-aspartate (NMDA)-dependent supralinear dendritic integration.^38^ This suggests that NMDA receptor activation could serve as a potential source of calcium entry, particularly during the spatiotemporally clustered synaptic inputs that would occur as seizures progress. Combined with their late-spiking profile and progressive recruitment from more distal regions, NMDA receptor activation may also contribute to the slow-onset activation of NDNF + cells.

Our findings thus suggest that feedforward somatic inhibition by PV+ neurons is followed by feedforward dendritic inhibition mediated by NDNF+ interneurons during epileptic activity. Previous observations suggest that SOM+ cell recruitment also lags behind that of PV+ neurons.^13^^,^^18^

The effect of optogenetic manipulation of NDNF+ cells contrasts with that of PV+ and SOM+ cells. Indeed, optogenetic depolarisation of NDNF+ interneurons persistently suppressed both interictal spikes and seizures, irrespective of the timing of stimulation. Whilst photo-depolarisation of NDNF+, PV+, and SOM+ cells initially suppresses interictal spiking activity, their activation induces an important rebound of activity by the end of stimulation. Consistently, whilst photo-depolarisation of all three interneuron classes at seizure onset exerts an anti-epileptic effect, their effects diverge markedly when depolarisation is delayed a few seconds into a seizure. NDNF+ cell activation elicits a persistent suppressive effect even when stimulated several seconds after seizure onset, whereas SOM+ cell excitation loses efficacy, and PV+ interneuron activation paradoxically exacerbates seizures.

Although the brain region, seizure model and optogenetic method were consistent across all experiments, several protocol differences must be acknowledged. PV+ and SOM+ cells are located in layers 2/3, 4, and 5, whereas NDNF+ cells are almost exclusively in layer 1.^6^^,^^7^^,^^71^ This makes it challenging to achieve identical illumination configurations across experiments. Optogenetic manipulations of PV+ and SOM+ cells were thus performed with fibres inserted into layer 2/3, whilst NDNF+ cell experiments were conducted with the fibre positioned above the cortex. Additionally, optogenetic depolarisation of PV+ and SOM+ cells used continuous light illumination, whereas NDNF+ cells were stimulated intermittently. However, the inhibitory charge transfer elicited by continuous or discontinuous stimulation patterns of PV+ cells is equivalent^21^ and the greatest charge transfer for NDNF+ cell stimulation occurs at high frequency, which approximates continuous stimulation conditions. Thus, whilst minor differences exist in the optogenetic experimental protocols, they are unlikely to be the primary cause of the observed differences.

Our findings thus underscore the importance of the interneuron-specific spatiotemporal dynamics of inhibitory restraint in an hyperexcitable environment with NDNF+ cells being more effective to maintain cortical excitability in check.

In the present study, we show that NDNF+ cells persistently suppress seizure activity and that blocking GABA_B_ receptors abolishes this anti-seizure effect. NDNF+ cells strongly inhibit both pyramidal neurons and PV+ cells (although not SOM+ cells).^6^^,^^44^ Consequently, activation of NDNF+ cells would be expected to also hyperpolarize PV+ cells during seizures, contributing to an anti-seizure effect given the paradoxical role of this interneuron type^21^^,^^28^ particularly when applied more than 2 s after seizure onset.^21^ This phenomenon may contribute to the persistent anti-seizure effect observed here. However, when GABA_B_ receptors are blocked, NDNF+ cell photo-depolarisation promotes seizure activity in a manner qualitatively similar to that observed with PV+ cells. In our previous study, we demonstrated that somatic inhibition mediated by PV+ cells becomes pro-epileptic as seizures progress and that this switch was at least partially due to GABA_A_ receptor mediated chloride loading of pyramidal cells.^21^ Chloride loading renders GABAergic inhibition at best inefficient and at worst depolarising.^73^, ^74^, ^75^ Whilst chloride loading presents an attractive explanation for the pro-seizure effect of NDNF+ cells under conditions of GABA_B_ receptor blockade, this hypothesis remains to be investigated. Indeed, other possibilities cannot be discounted, such as chloride loading due to GABA_A_ receptor activation leading to potassium efflux and depolarisation of neighbouring neurons.^76^, ^77^, ^78^

Our findings thus suggest that GABA_B_-mediated inhibition is critical for the suppressive role of NDNF+ interneurons under epileptic conditions.

A relatively subtle increase in NDNF+ cell excitability by hM3Dq activation yielded an important influence on cortical network excitability in both extreme physiological and pathological conditions. Although preliminary, these results suggest that enhancing the inhibitory function of NDNF+ cells could represent a viable therapeutic strategy for preventing seizures in TLE. Notably, NDNF+ neurons exhibit better survival in pathological conditions compared to PV+ and SOM+ cells.^64^, ^65^, ^66^, ^67^, ^68^, ^69^ Importantly, the NDNF marker is conserved in human NGF cells,^79^ which could facilitate the translation of these findings into clinical applications. This is particularly relevant given the ongoing efforts in gene therapy for pharmaco-resistant epilepsy.^3^^,^^4^^,^^80^ NDNF+ cells also express neuropeptide Y (NPY), which has intrinsic anti-seizure properties^47^^,^^81^^,^^82^ and could therefore contribute to the anti-seizure effect mediated by NDNF+ cell activation. However, much more work is needed to understand whether NDNF+ cells can release NPY and under which condition.

Additionally, our data highlight the critical role of GABA_B_ receptor activation in restraining hyperexcitation. Whilst GABA_B_ receptor agonists have shown deleterious effects in absence epilepsy,^83^ accumulating evidence suggests their potential as a therapeutic target in refractory partial epilepsy. GABA_B_ receptor agonists have demonstrated anti-seizure effects in various preclinical models of epilepsy,^63^^,^^84^^,^^85^ and our findings provide further support for this approach. Harnessing NDNF+ cell-mediated inhibition could offer the additional advantage of recruiting GABA_B_ receptors locally and only when needed, specifically during intense neuronal activity, reducing the potential for receptor desensitisation.

Whilst our study provides evidence that NDNF+ cells and GABA_B_ receptor activation are promising targets for treating refractory epilepsy, it is by nature exploratory. In addition, most of our data derive from acute epilepsy models, and while we obtained some encouraging preliminary data in a chronic model of TLE, we had only a limited number of animals. Investigating the potential anti-seizure effects of NDNF+ cells in a pre-clinical study fully powered as well as in other chronic epilepsy models and across different species would be essential to firmly establish this NDNF+ cell activation approach to treat epilepsy. Additionally, NDNF+ cells and GABA_B_ receptors have been shown to strongly regulate cortical and hippocampal microcircuit activity under physiological conditions.^6^^,^^10^^,^^37^^,^^44^^,^^71^^,^^85^, ^86^, ^87^, ^88^ Their manipulation could therefore have significant off-target effects on cognitive processes, necessitating further preclinical investigation into the effects of chemogenetic activation of NDNF+ cells on learning and memory in both healthy and epileptic animals.

Collectively, our results highlight NDNF+ cells and GABA_B_ receptor-mediated signalling as candidate anti-seizure targets for the treatment of temporal lobe epilepsy, and call for further evaluation in complementary preclinical models.

## Contributors

VM, AR, MSM, DMK designed the experiments. VM, AR, MSM, YS, AG, MM, SAB, MG, QW performed the experiments. VM, AR, DMK, RTG, ML analysed the data. AR, MM, AL designed and produced the various plasmids. VM, AR, DMK wrote the manuscript, and all the authors revised it. VM and AR had accessed and verified all the data. VM, DMK secured funding. All authors have read and approved the final version of the manuscript.

## Data sharing statement

The datasets generated and analysed during this study are available from the corresponding authors upon reasonable request (email: vincent.magloire@inserm.fr; amy-richardson@ucl.ac.uk; d.kullmann@ucl.ac.uk). PyECoG software is available at: https://github.com/KullmannLab/pyecog2.

## Declaration of interests

A Richardson has consulted for EpilepsyGTx Ltd.. M Leite has consulted for UCB Pharma. D Kullmann has consulted for, and has equity in, EpilepsyGTx. M Muller is employed by SynapCell SAS. None of these commercial entities had any role in the funding, design or execution of this study. All the other authors declare no conflict of interest.
