# Supplementary material for: Neuron-derived neurotrophic factor-positive interneurons: a cellular target for anti-seizure therapies
Source: eBioMedicine. 2026 Jul 21;130:106362. doi: 10.1016/j.ebiom.2026.106362 (PMC13393760; doi:10.1016/j.ebiom.2026.106362)
Supplement: Supplementary Figs. S1–S9 [file mmc1.docx]

Supplementary figures:


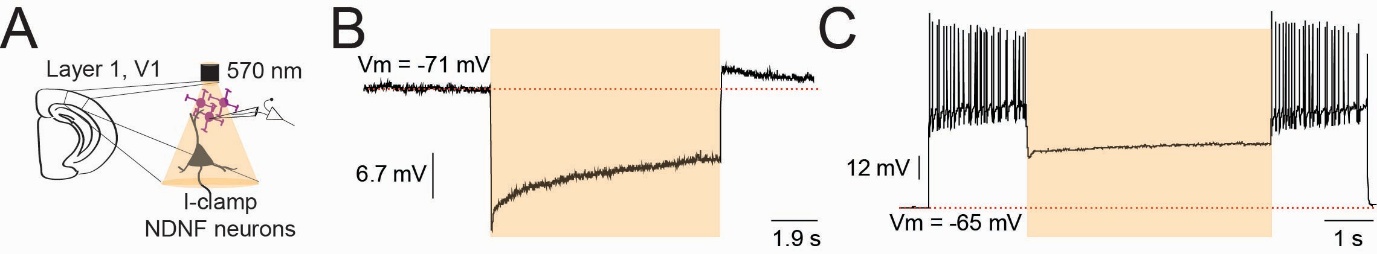


**Fig. S1: NDNF+ cell optogenetic hyperpolarisation with mDlx-FLEX-ArchT-GFP.** (**A**) Experimental setup. (**B**) Example of light-induced membrane hyperpolarisation recorded from an NDNF+ neuron expressing ArchT-GFP from a resting potential of -71 mV. Yellow shaded area 10s-long illumination. (**C**) Example of voltage trace from an NDNF+ neuron during injection of a depolarising current step to elicit action potentials. Laser activation (yellow shaded area) inhibited firing.


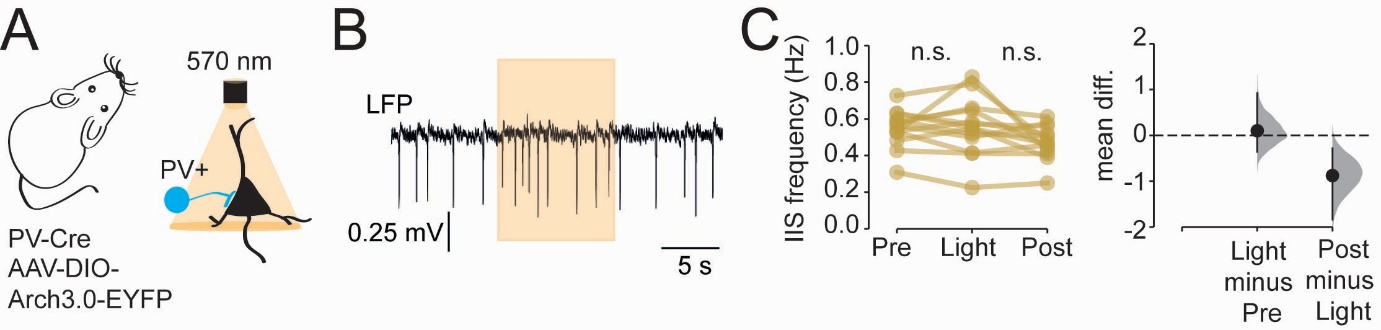


**Fig. S2: Effect of optogenetic hyperpolarisation of PV + cells on interictal spiking.** (**A**) Experimental setup. (**B**) Example of LFP trace showing interictal spiking following pilocarpine injection. Illumination of PV neurons with 570 nm light (yellow shaded area, 10 s) led to a small increase in interictal spiking. (**C**) Overall, the interictal spike frequency across experiments did not change with light activation and exhibited a borderline-significant subsequent decrease (one-way repeated ANOVA followed by Sidak post-hoc test, the mean difference between Pre and Light is 0.01 [95%CI, -0.04, 0.09], p = 0.93; between Post and Light -0.09 [95%CI, -0.18, -0.03], p = 0.09; effect size (d): 0.33, n= 13 mice). Error bars correspond to the 95% confidence intervals.


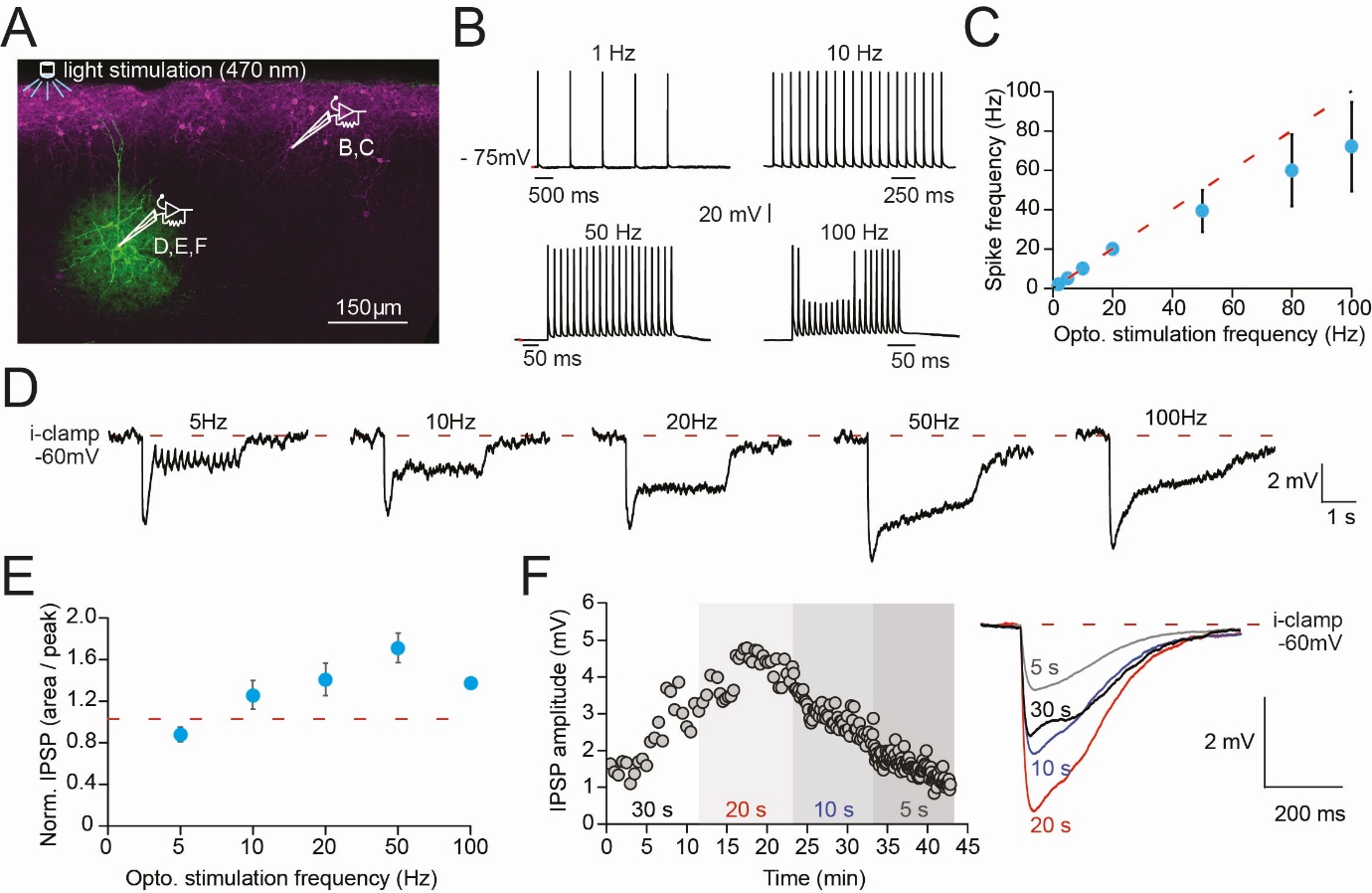


**Fig. S3: Optogenetic stimulation of NDNF+ cells: exploration of parameter space.** (**A**) Chronos-GFP expression in NDNF+ neurons in layer 1 of visual cortex (magenta) together with a patched pyramidal neuron loaded with biocytin (green), scale bar: 150 µm. (**B**) Current clamp recordings from NDNF+ neuron expressing Chronos in response to 470 nm laser stimulation at different frequencies. (**C**) NDNF+ neuron firing frequency plotted against laser pulse frequency (n=4 cells). (**D**) Current clamp recordings from a neighbouring pyramidal neuron. (**E**) IPSP area normalised to peak hyperpolarization in response to optogenetic NDNF+ cell stimulation at different frequencies (n=7 cells). (**F**) Amplitude of IPSP in response to 1 ms light pulses every 30 seconds, 20 s, 10s or 5s. Error bars correspond to the SEM.


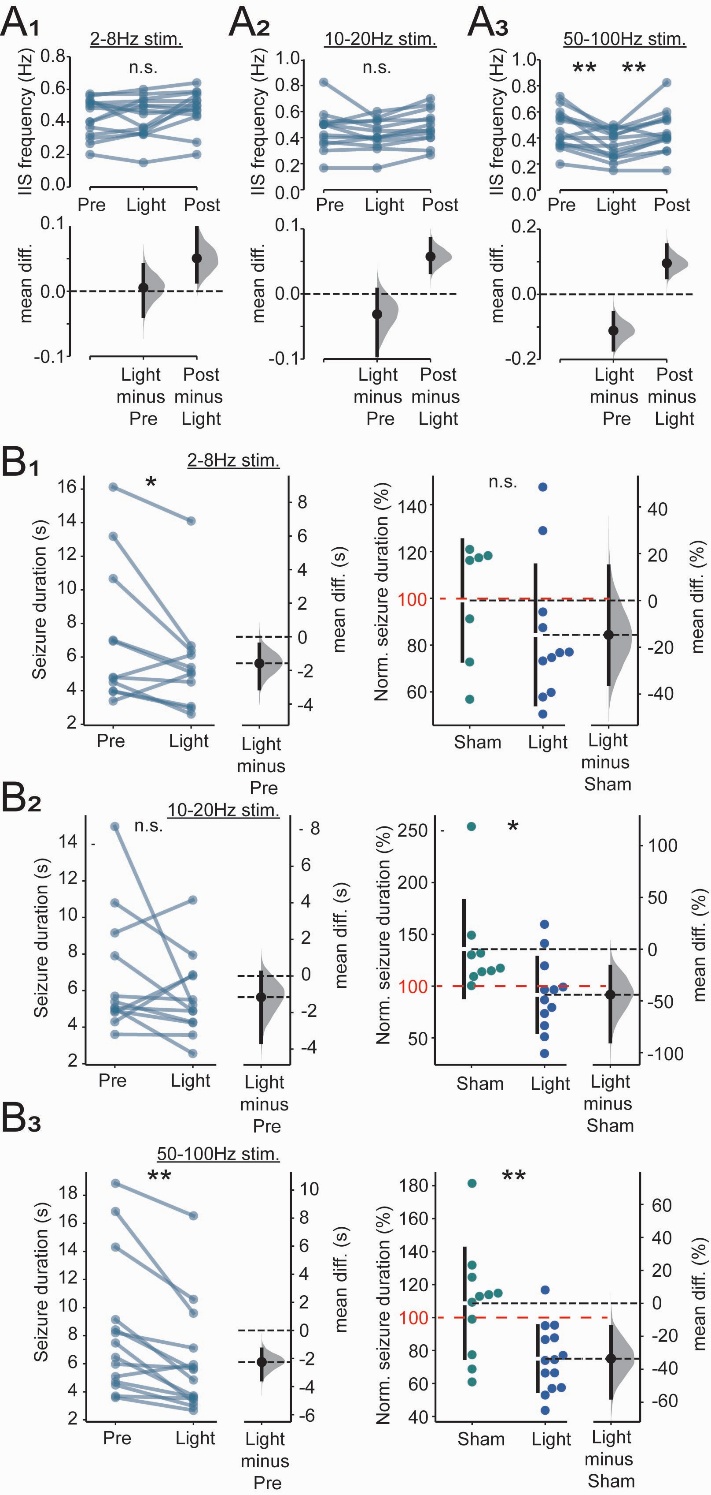


**Fig. S4:** **Frequency dependence of cortical network inhibition by optogenetic NDNF+ neuron depolarisation*.*** (**A**) Interictal spiking following pilocarpine injection was unchanged in mice expressing Chronos-GFP in NDNF+ neurons when laser pulses were delivered at 2-8 Hz (one-way repeated ANOVA followed by Sidak post-hoc test; mean difference between Pre and Light is 0.005 [95%CI, -0.038, 0.040], p=0.95; mean difference between Post and Light is 0.05 [95%CI, 0.02, 0.10], p=0.08, effect size (d): 0.22, n=14 mice, A_1_). When pulses were delivered at 10-20Hz, a non-significant decrease in spiking was observed, (one-way repeated ANOVA; mean difference between Pre and Light is -0.03 [95%CI, -0.10, 0.007]; mean difference between Post and Light is 0.06 [95%CI, 0.03, 0.08], effect size (d): 0.18, n=13 mice, A_2_). Optogenetic stimulation at 50-100 Hz yielded a significant decrease in interictal spike frequency (one-way repeated ANOVA followed by Sidak post-hoc test; mean difference between Pre and Light is -0.11 [95%CI, -0.17, -0.06], p=0.005; mean difference between Post and Light is 0.09 [95%CI, 0.05, 0.15], p= 0.006, effect size (d): 0.35, n=14 mice, A_3_). (**B**) Seizure duration exhibited a borderline-significant decrease when laser activation, triggered by the sentinel spike, was delivered at 2-8 Hz (the mean difference between Pre and Light is -1.58 s [95%CI, -3.08, -0.45], p=0.041, effect size (d): 0.41, two-sided permutation t-test, n=11 mice; mean difference between Sham and Light is -14.7% [95%CI, -35.8, 14.6], p=0.29, effect size (d): 0.52, two-sided permutation t-test, Sham n= 7 mice, Light n=11 mice, B_1_). Similar results were obtained at 10-20Hz (the mean difference between Pre and Light is -1.16 s [95%CI, -3.63, 0.19], p=0.26, effect size (d): 0.39, two-sided permutation t-test, n=12 mice; mean difference between Sham and Light is -44.0% [95%CI, -89.3, -16.8], p=0.013, effect size (d): 1.06, two-sided permutation t-test, Sham n= 9 mice, Light n=12 mice, B_2_). Laser activation at 50-100Hz yielded a robust decrease in seizure duration (the mean paired difference between Pre and Light is -2.25 s [95%CI, -3.51, -1.34], p=0.001, effect size (d): 0.50, two-sided permutation t-test, n=14 mice; Unpaired mean difference between Sham and Light is -33.5% [95%CI, -57.3, -14.3], p=0.003, effect size (d): 1.22, two-sided permutation t-test, Sham n= 11 mice, Light n=14 mice, B_3_). Error bars correspond to the 95% confidence intervals.


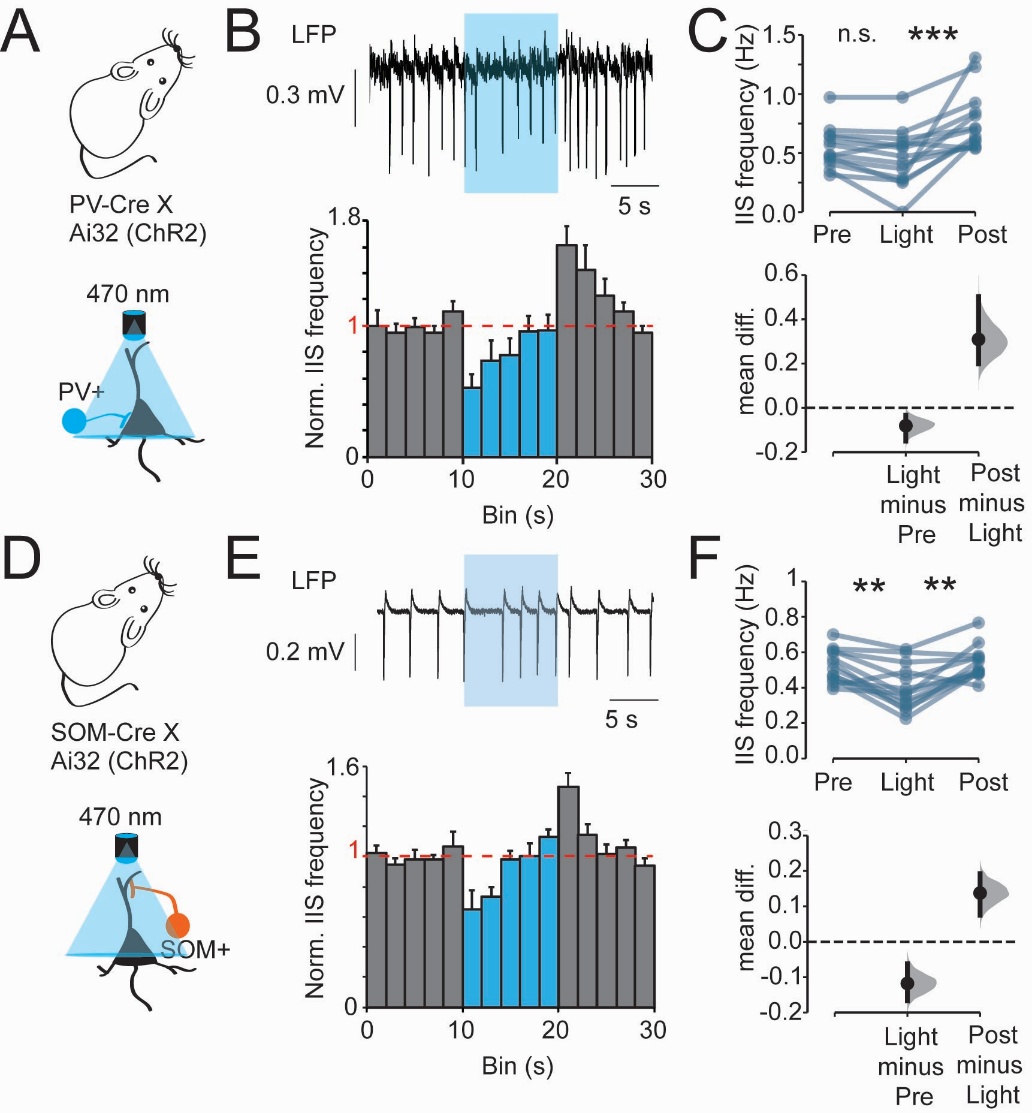


**Fig. S5: Effect of optogenetic depolarisation of PV+ and SOM+ cells on interictal spiking activity.** (**A**) Experimental setup for PV+ cell stimulation. (**B**) Representative LFP trace from a PV-Cre mouse showing interictal spiking following pilocarpine injection (Top panel). The blue box indicates time of light exposure. (Bottom panel) Normalised mean IIS frequency overall before, during and after light exposure (binning window 2s). Error bars correspond to the SEM. (**C**) Interictal spiking was modestly decreased during light stimulation compared to a preceding 10 s period. Interictal spiking was increased following cessation of light stimulation compared to the illumination period. (Friedman test followed by a post-hoc Dunn’s test; mean difference between Pre and Light is -0.08 Hz [95%CI, -0.15, -0.03], p= 0.14; mean difference between Post and Light is 0.31 [95%CI, 0.20, 0.50], p<0.001; effect size (d): 1.52, n=13 mice). Error bars correspond to the 95% confidence intervals. (**D**) Experimental setup for SOM+ cell stimulation. (**E**) Example LFP trace from a SOM-Cre mouse (Top panel), (Bottom panel) Normalised mean IIS frequency overall before, during and after light exposure (binning window 2s) as in (B). Error bars correspond to the SEM. (**F**) Interictal spiking was decreased during light stimulation (one-way repeated measures ANOVA followed by Sidak post-hoc test; mean difference between Pre and Light is -0.12 Hz [95%CI, -0.17, -0.06], p=0.002; mean difference between Post and Light is 0.14 [95%CI, 0.07, 0.19], p=0.001, effect size (d): 0.59, n=13 mice). Error bars correspond to the 95% confidence intervals.


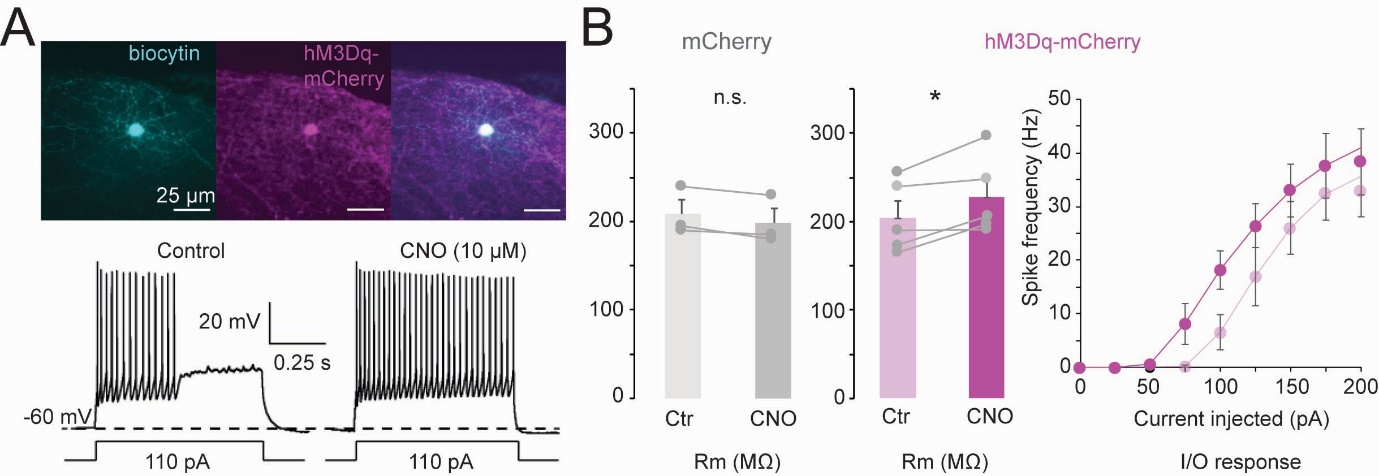


**Fig. S6: Activation of hM3Dq increases NDNF+ cell excitability ex vivo.** (**A**) Example NDNF+ neuron labelled with hM3Dq-mCherry (purple) that was patched and loaded with biocytin (cyan), scale bar: 25 µm. (Bottom panel) Example current clamp recordings from the labelled neuron above. Action potentials elicited by depolarising current injection before and after application of CNO. (**B**) Input resistance in NDNF+ neurons expressing either mCherry only (grey) or hM3Dq-mCherry (purple) before and after addition of CNO. CNO significantly increased the input resistance in neurons expressing hM3Dq (paired t-test, p=0.038, effect size (d): 0.54, n=5 neurons). (Right panel*)* Spike frequency in response to increasing current step injections before (light purple) and after (dark purple) addition of CNO. Error bars correspond to the SEM.


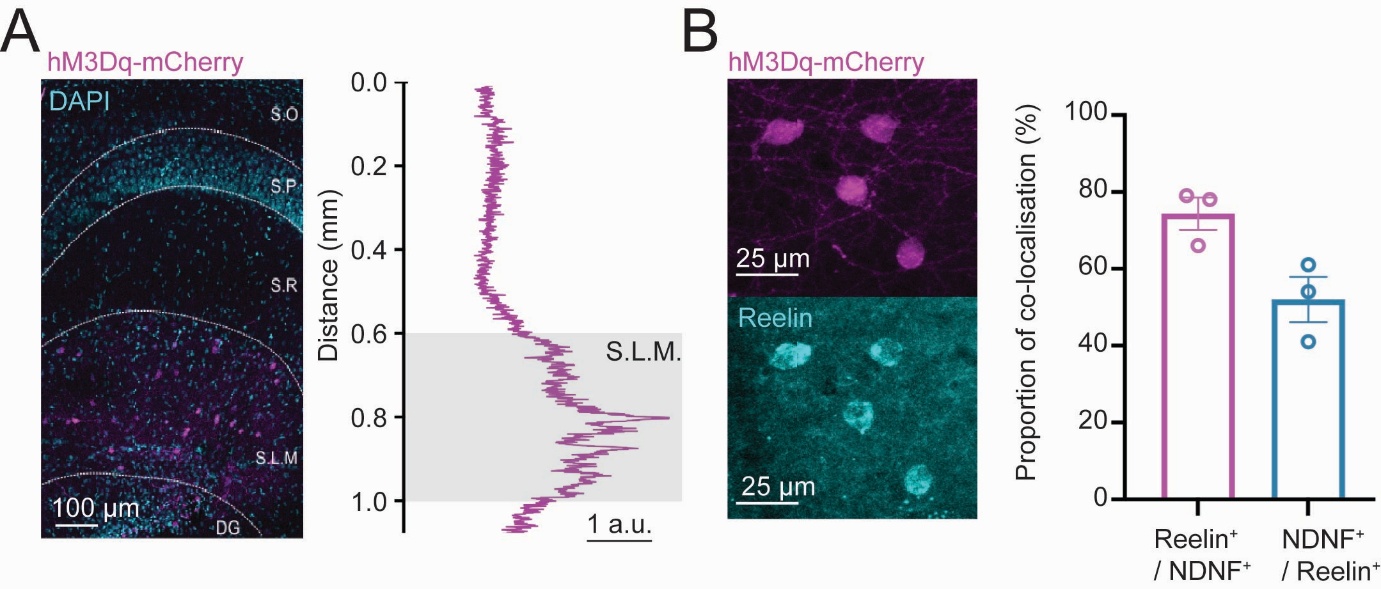


**Fig. S7: NDNF+ cell localisation and molecular profile in the hippocampus.** (**A**) mCherry expression (magenta) and DAPI staining (cyan) in the hippocampus of an NDNF-Cre mouse injected with AAV9-mDlx-FLEX-hM3Dq-mCherry. mCherry expression was restricted to the Stratum Lacunosum Moleculare (S.L.M) of the hippocampus. (Right panel*)* Fluorescence profile of mCherry staining throughout the layers of the hippocampus. Fluorescence was concentrated in the S.L.M. Abbreviations: S.O = Stratum Oriens, S.P = Stratum Pyramidale, S.R = Stratum Radiatum, DG = Dentate Gyrus. (**B**) mCherry expression (magenta) in NDNF+ neurons in the S.L.M co-stained with the NGF cell marker Reelin (cyan), scale bar: 25 µm. The mean proportion of mCherry-positive cells expressing reelin was 74 ± 4.2% and the mean proportion of reelin cells expressing mCherry was 52 ± 5.5%, n = 3 mice, 3 slices per animal. Error bars correspond to the SEM.


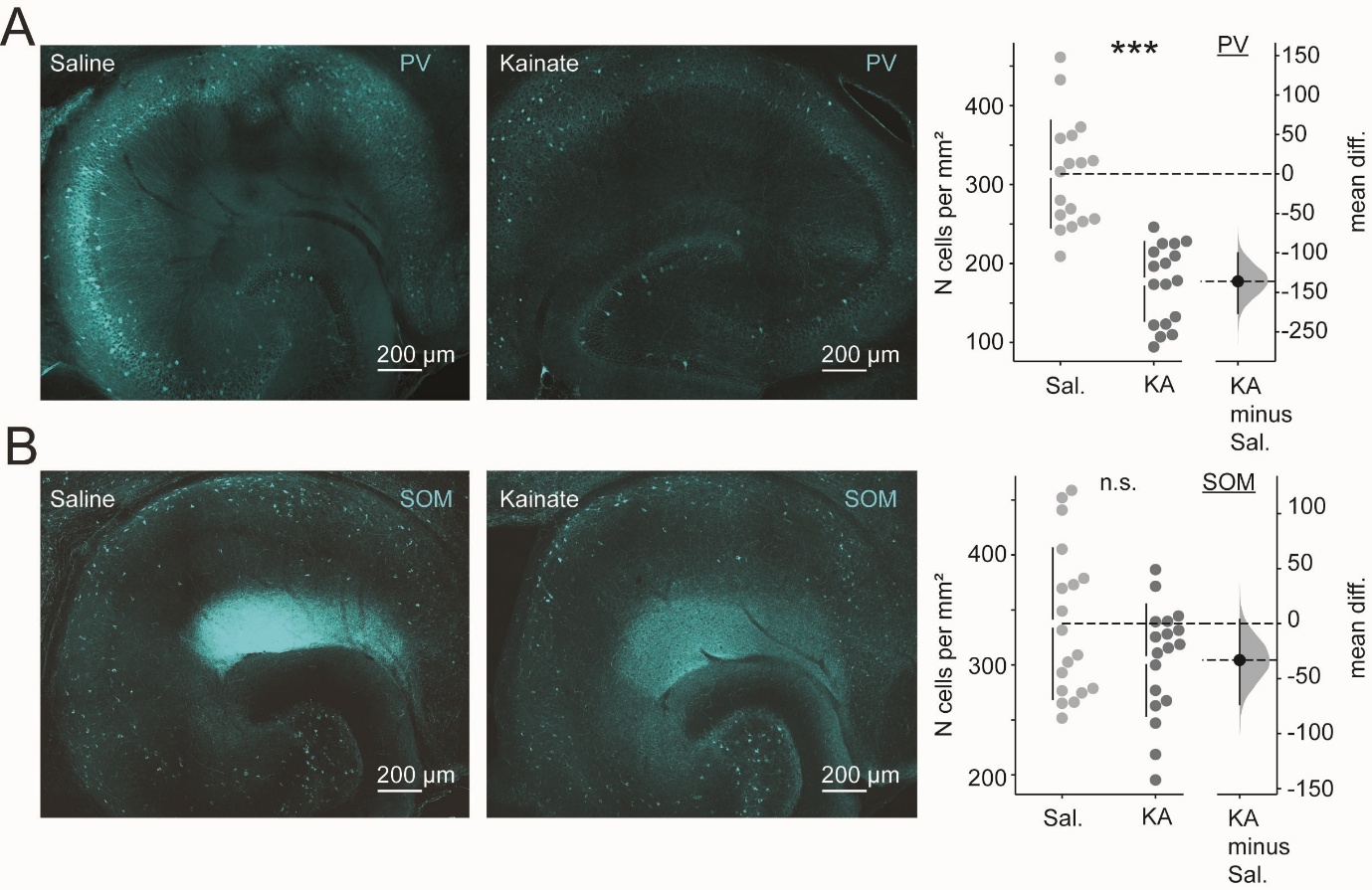


**Fig. S8: Loss of hippocampal interneurons in the intra-hippocampal kainate model of temporal lobe epilepsy.** (**A**) Representative images of parvalbumin (PV) staining in the hippocampus of a mouse injected with intrahippocampal saline (left) or kainate (right), scale bar: 200 µm. The number of PV+ neurons was reduced in epileptic mice injected with intrahippocampal kainate (n=18 slices / 3 animals) compared to non-epileptic controls (n= 18 slices/ 3 animals; mean difference is -136 cells [95%CI, -176, -100], p<0.001, effect size (d): 2.28, two-sided permutation t-test). (**B**) Somatostatin (SOM) staining in the hippocampus of a non-epileptic saline injected control (left) or a kainate-injected epileptic mouse (right), scale bar: 200 µm. The number of SOM+ interneurons was slightly reduced in epileptic mice (n = 18 slices / 3 mice) compared to saline-injected controls (n = 18 slices / 3 mice, mean difference is -33 cells [95%CI, -73, 4], p=0.11, effect size (d): 0.55, two-sided permutation t-test). Error bars correspond to the 95% confidence intervals.


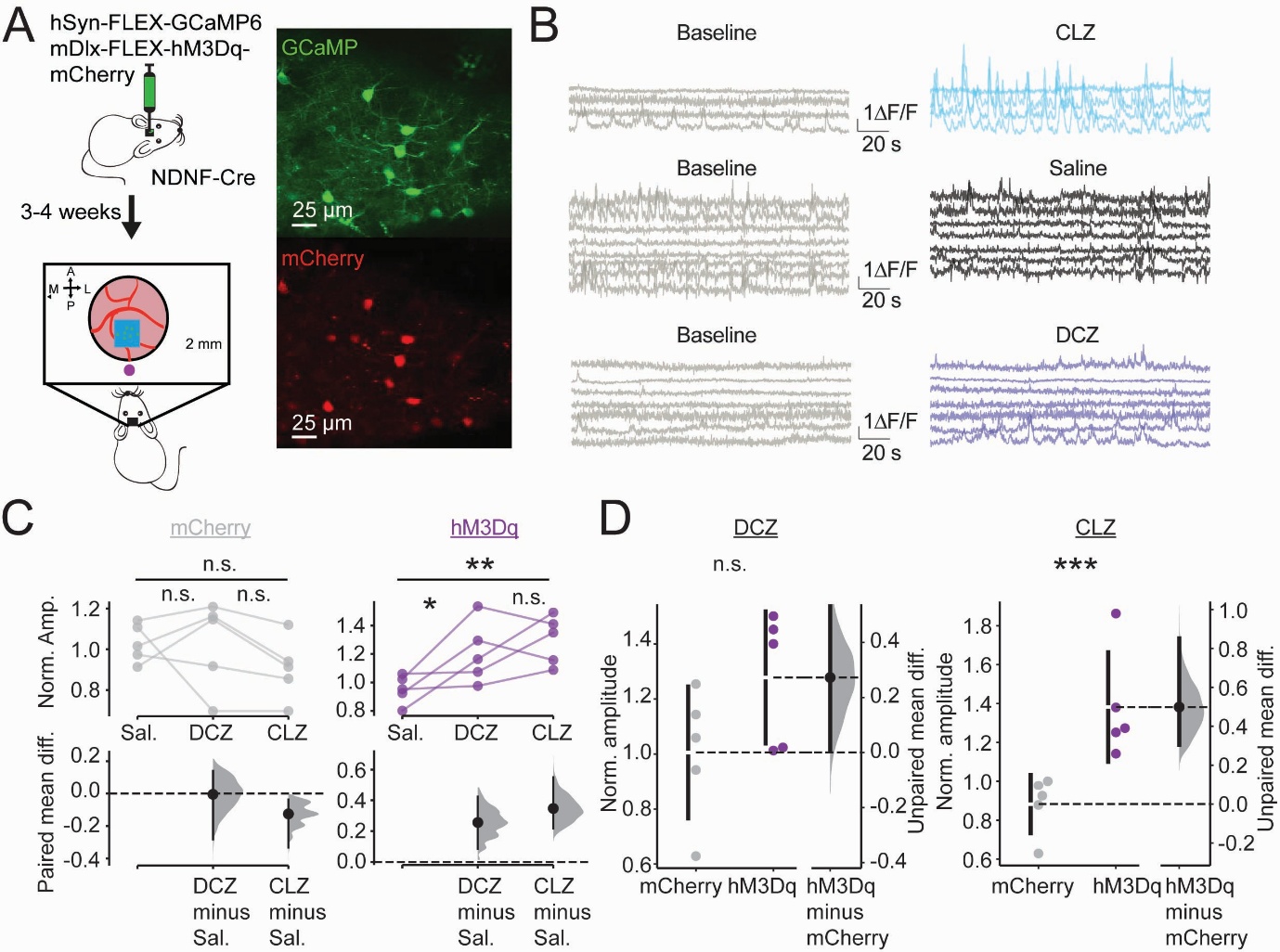


**Fig. S9: In vivo chemogenetic activation of NDNF+ cells.** (**A**) Schematic of experimental setup. (Right panel) Example GCaMP6f expression (green) and mCherry expression (red) in NDNF+ neurons within a field of view, scale bar: 25 µm. (**B**) Representative calcium traces from a single mouse before (baseline) and after injection of clozapine (CLZ; blue), saline (dark grey) and deschlorclozapine (DCZ; purple). (**C**) Average amplitude of calcium transients following injection of DCZ, saline or CLZ normalised to the baseline period. In mice expressing mCherry only (grey) no change in amplitude was observed in the presence of DCZ or CLZ compared to saline (paired mean difference between Saline and DCZ is 0.004 [95%CI, -0.15, 0.24], paired mean difference between CLZ and Saline is -0.12 [95%CI, -0.33, -0.04], effect size (d): 0.53, n= 5 mice). In animals expressing hM3Dq-mCherry, an increase in amplitude of calcium transients was seen with DCZ and CLZ compared to saline (mean difference between Saline and DCZ is 0.26 [95%CI, 0.09, 0.42]; mean difference between CLZ and Saline is 0.34 [95%CI, 0.21, 0.55], two-way repeated measures ANOVA, Treatment x Virus, p=0.007, post-hoc Tukey test, hM3Dq group, saline vs DCZ, p=0.030, saline vs CLZ, p=0.003, DCZ vs CLZ, p=0.583, effect size (d): 0.53, n= 5 mice). (**D**) Amplitude of calcium transients following injection of DCZ (left, mean difference between mCherry and hM3Dq is 0.27 [95%CI, 0.01, 0.55], p=0.10, effect size (d): 1.14, two-sided permutation t-test, n=5 mice per group) and CLZ (right, mean difference between mCherry and hM3Dq is 0.49 [95%CI, 0.30, 0.85], p<0.001, effect size (d): 2.21, two-sided permutation t-test, n=5 mice per group) normalised to baseline. Error bars correspond to the 95% confidence intervals.
